# Targeting the SARS-CoV-2 HR1 with Small Molecules as Inhibitors of the Fusion Process

**DOI:** 10.3390/ijms231710067

**Published:** 2022-09-03

**Authors:** Davide Gentile, Alessandro Coco, Vincenzo Patamia, Chiara Zagni, Giuseppe Floresta, Antonio Rescifina

**Affiliations:** Dipartimento di Scienze del Farmaco e Della Salute, Università di Catania, Viale A. Doria 6, 95125 Catania, Italy

**Keywords:** SARS-CoV-2, docking simulations, molecular dynamics simulations, pharmacophore modeling

## Abstract

The rapid and global propagation of the novel human coronavirus that causes severe acute respiratory syndrome coronavirus 2 (SARS-CoV-2) has produced an immediate urgency to discover promising targets for the treatment of this virus. In this paper, we studied the spike protein S2 domain of SARS-CoV-2 as it is the most conserved component and controls the crucial fusion process of SARS-CoV-2 as a target for different databases of small organic compounds. Our in silico methodology, based on pharmacophore modeling, docking simulation and molecular dynamics simulations, was first validated with ADS-J1, a potent small-molecule HIV fusion inhibitor that has already proved effective in binding the HR1 domain and inhibiting the fusion core of SARS-CoV-1. It then focused on finding novel small molecules and new peptides as fusion inhibitors. Our methodology identified several small molecules and peptides as potential inhibitors of the fusion process. Among these, NF 023 hydrate (MolPort-006-822-583) is one of the best-scored compounds. Other compounds of interest are ZINC00097961973, Salvianolic acid, Thalassiolin A and marine_160925_88_2. Two interesting active peptides were also identified: AP00094 (Temporin A) and AVP1227 (GBVA5). The inhibition of the spike protein of SARS-CoV-2 is a valid target to inhibit the virus entry in human cells. The discussed compounds reported in this paper led to encouraging results for future in vitro tests against SARS-CoV-2.

## 1. Introduction

Several coronaviruses have spread in the past, such as HCoV-229E, SARS-CoV-1 (2002), HCoV-OC43, MERS-CoV (2012), HCoV-HKU1 and HCoVNL63 [1,2,3,4,5]. In December 2019, an outbreak of severe pneumonia of unknown etiopathology in Wuhan, China was recognized by the World Health Organization (WHO). Later genetic sequencing of the responsible virus showed that the responsible microorganism was a positive-sense single-stranded RNA virus belonging to β-coronavirus lineage B (SARS-CoV 79.5% genome identity). The WHO named the virus SARS-CoV-2 and declared the outbreak a pandemic [6,7].

The new coronavirus penetrates the respiratory system, causing different symptoms, and the gravity of the resulting inflammation is correlated with the health states and ethnicities of the patients [2,8]. The current data (https://www.worldometers.info/coronavirus/, accessed on 22 July 2022) count 572,887,455 cases, 6,398,672 deaths and 542,886,946 recovered.

Various studies have been focused on repurposing approved antiviral drugs, such as Remdesivir [9], and antibodies targeting the spike–ACE2 interfaces have also been reported [10]. Meanwhile, the lack of a specific COVID-19 drug is still a problem, and vaccination has been proven to be the main route for prevention [11].

Coronaviruses contain four structural proteins named envelope (E), spike (S), membrane (M) and nucleocapsid (N). SARS-CoV-2 relies on its spike protein to enter human cells [12]. The mechanism of entrance and fusion is relative to the homotrimeric transmembrane S protein. The fusion process requires high energy to contrast the repulsive forces of the membranes; this energy is generated by the fusion protein’s refolding and its conformational change [13]. In the prefusion state, the spike protein has a central trimeric structure surrounded by other fragments. CH, CD and HR2 domains form the central three-stranded coil.

Conformational change leads to a post-fusion state where the HR2 domain binds to HR1, forming an antiparallel six-helix bundle (6-HB). This conformation acts as a spring that brings the virus membrane to fusion with the host cell membrane and allows the uncoating process with the release of the viral RNA into the host cell cytoplasm. The 6-HB domain presents a hydrophobic pocket before the TM domain; this seems to be conserved over several coronaviruses.

HR1 and HR2 are part of the S2 subunit, together with the fusion peptide (FP), the transmembrane region (TM) and the cytoplasmatic tail (CT). The residues of the central three-stranded coil involved in the fusion process represent the fusion core that extends approximately from residue 920 of the HR1 domain to residue 960. SARS-CoV-2 shares with SARS-CoV-1 92.6% identity for the HR1 region and 100% for HR2. Both share a central portion with hydrophobic residues that help the interaction with HR2, which binds the side of two HR1 domains. Moreover, nine residues (A1174, V1177, N1178, Q1180, E1182, N1194, E1195, S1196 and L1197) from the HR2 domain create hydrogen interactions with HR1 [13,14,15].

SARS-CoV-2 seems to have better membrane fusion capacity than SARS-CoV-1, enhancing the potential effect of fusion inhibition for this coronavirus. Eight different residues justify the differences in the enhanced fusion ability [16].

Old research focused on finding valuable peptides inhibitors of the fusion process against coronaviruses. Among these, the EK1 peptide was found to be a good candidate, and it was also crystallized with the HR1 domain of SARS-CoV-1 [14]. This peptide has already been tested for SARS-CoV-1 [17] and MERS-CoV [18] and reported as an inhibitor of the fusion core. EK1 has also been tested for SARS-CoV-2, showing an inhibitory effect in a dose-dependent manner [19]. Nowadays, different peptides are being tested to find better inhibition results, such as IPB01 and IPB02, which showed increased efficacy, confirmed by in vitro assays against SARS-CoV-2 with IC_50_ values of 0.022 and 0.025 μM, respectively [20]. Further optimization of the structure of the EK1 peptide has been performed, and new screening and molecular dynamics (MD) led to other exciting peptides [21,22].

As with SARS-CoV-1, even HIV-1 studies have involved the usage of peptides to interrupt fusion core formation. Enfuvirtide was approved by the US FDA for HIV-1 treatment, confirming its gp41 inhibitory activity; the peptide has been largely used for combination therapy [23]. Recently, some peptides and some small molecules have been reported to bind the HR1 domain and inhibit the fusion core of SARS-CoV-1. Among these molecules, ADS-J1 showed the highest activity. In vitro testing showed that ADS-J1 interferes with the binding of HR2 to HR1. Moreover, the X-ray crystal structure shows a deep pocket in the SARS-CoV HR1 surface (Phe909 to Leu927). ADS-J1 has been studied in a docking experiment in a rigid pose so that its hydrophobic region could bind to the hydrophobic pocket on the surface of HR1 with favorable energy [24].

Nowadays, in silico medicinal chemistry has generated major interest in the research field, leading to significant results, especially in drug repurposing, generally reducing the time and cost of developing novel pharmaceuticals. In fighting COVID-19 [25,26,27,28,29,30,31,32,33,34,35], the effectiveness of artificial intelligence approaches has also been demonstrated in addition to the more classic computational studies based on ligands and structures [36]. Since several successes in this field have proved the validity of such tools, our research group has gained experience over the years in the field of computational drug design [37,38,39,40,41,42,43].

In this paper, we decided to build a pharmacophore model based on the post-fusion core of the S2 subunit (PDB ID: 6LXT) [16] and exploit it to evaluate several libraries of small molecules and peptides to find novel inhibitors of the fusion process. The process is resumed in Scheme S1, especially for small molecules. We first screened the whole dataset using a pharmacophore model, and the best compounds were docked and analyzed through MD simulations. For peptides, the selection was based on the HDOCK server, and the hit compounds and peptides were evaluated by MD simulations. Nine small molecules and two peptides were identified as high-scoring compounds in the inhibition of the fusion process.

## 2. Results and Discussion

### 2.1. Pharmacophore-Based Virtual Screening

The pharmacophore-based virtual screening is based on the HR1–HR2 complex pharmacophore model shaped using the Pharmit server (http://pharmit.csb.pitt.edu, accessed on 1 June 2022).

The chemical structures databases used for the virtual screening (VS) were FDA-approved drugs, marine natural products (MNP), MolPort, ZINC, ChemSpace, PubChem, CHEMBL25 and supernatural products II Marvin (MNP), for a total of more than 166,149,376 molecules and 960,875,860 conformers.

The main selected interactions from the HR1–HR2 complex model were: the hydrogen bond donor interaction Ser1196-Ser929, the strong hydrophobic interactions for Leu1193 and Leu1186 with the HR1 pocket (involving Ala930, Ile931, Leu938 and Ala942) and the hydrogen bond acceptor interactions for Asp1194-Gln935, Asp1192-Lys933, Gln1182-Lys947 and Gln1180-Gln949. We found that the interactions of ADS-J1 with HR1 are in common with the selected interactions that occur within the complex HR1–HR2. Therefore, we expect that ligands obtained from PBVS show hydrophobic interactions with the central portion of HR1 and hydrogen bonds with the terminal parts of the molecules.

We then performed an analysis for the pharmacophore-based model using EK1 peptide synthesized as a fusion inhibitor for SARS-CoV-2, which, earlier, showed a high affinity, with an IC_50_ of 0.2 µM (PDB ID: 7C53) [44].

The results of the calculations showed that there are: hydrophobic interactions at the interface of the two HR1 loops, especially in the central pocket, a hydrogen bond donor interaction for Ser1029 with Ser929, plus hydrogen bond acceptor interactions for Tyr1030 and Glu1027 residues interacting in the binding site with a residue of Gln935. Moreover, there are other relevant interactions between the two molecules, such as Leu1026 with Lys933, Glu1013 with Gln949 and Glu1021 with Lys947, and charge–charge interaction for Glu1021 and Glu1028 with Lys947 and Lys933.

These results evidenced that the main interactions of EK1 with the HR1 domain are in common with the interactions of the HR2 domain in the fusion core complex (Supplementary Material). This analysis again confirms the main selected interactions for Heptad Repeat Domain 1 and 2 in forming the six-helix bundle core. Therefore, we have ensured that our pharmacophore model is based on the HR1–HR2 complex. The full details with database name, number of molecules, number of conformers, score cutoff and molecules that passed the pharmacophore filter are reported in Table 1.

The remaining compounds have been selected, discarding large molecules and glycol-derived structures, which will hardly maintain stable interactions at the binding site over time. The selected compounds have been analyzed with docking simulation.

### 2.2. ADS-J1’s Docking and MD Simulation

Re-docking for ADS-J1 with SARS-CoV-1 fusion core (PDB ID: 1WNC) [24] was performed to validate its activity and confirm the type of interactions. Furthermore, we also tested ADS-J1 for the SARS-CoV-2 fusion core (PDB ID: 6LXT).

The pose selected for SARS-CoV-1 shows significant interactions, with an affinity of −9.76 kcal/mol. The molecule engages a network of hydrogen bonds. It makes two bonds with donor residues Gln931, one with a nitrogen atom from the diazonium bridge and another with an oxygen atom from the sulfonic acid group of the naphthalene (2.91 Å and 1.81 Å); Ser924 interacts with the nitrogen of the triazine (1.86 Å), Gln917 relates with the nitrogen from the other diazonium bridge (with a distance of 2.17 Å and a D-H-A angle of 149.25°) from helix C and Gln908 in helix A makes two bonds with two oxygen from sulfonic acid (2.73 Å, 1.81 Å); Lys929 in helix A interacts with the oxygen atoms of a terminal sulfonic acid (1.82 Å).

Lys911 and Lys929 anchor the compound through salt bridges and makes other interactions with sulfonic acid groups (Lys911 establishes two attractive charge interactions of 5.35 Å and 5.36 Å, while Lys929 makes one salt bridge 1.83 Å). Lys911 also makes a *π*–cation interaction with the naphthalene group. There are also other interactions, such as van der Waals forces, carbon–hydrogen bonds and alkyl plus *π*–alkyl interactions. Lastly, in the central part of the ligand, aryl rings occupy the hydrophobic zone, with the phenyl group linked to triazine, which plays an essential role in hydrophobic interaction, with Leu920 going deep in the pocket and a terminal aryl sulfonic ring taking place between Lys929 of helix A and Gln931 of helix C.

For the SARS-CoV-2 complex, we found that the best pose of interactions for ADS-J1 had a calculated docking binding energy of –8.8 kcal/mol. This pose is very similar to ADS-J1 interaction with SARS-CoV-1. It establishes H-bond interactions between the methoxy group and Lys947 (two interactions 2.48 Å and 2.74 Å) and *π*–alkyl interactions and *π*–cation interactions for Lys947 with the two naphthalene groups. Alkyl interactions further occur with Leu938, Ala942 and Lys933.

In both SARS-CoV-1 and SARS-CoV-2, two positive charged residues, Lys933 and Lys947, are engaging salt bridges and attractive charge interactions with negatively charged sulfonic acid (Lys947 makes a charge interaction and salt bridges 4.42 Å and 1.85 Å and Lys933 one bridge, 1.63 Å). ADS-J1, also for SARS-CoV-2, inserts its phenyl group in the hydrophobic pocket, interacting with Ser937, Leu938, Thr941 and Ala942.

**Figure 1:**
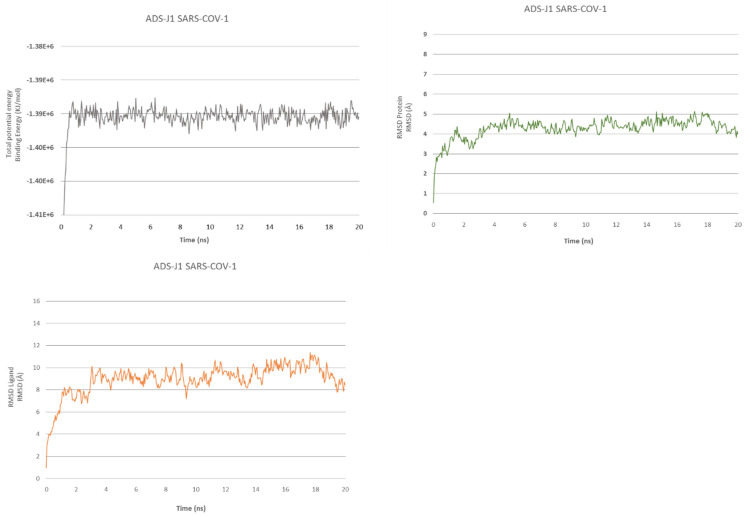
ADS-J1@SARS-CoV-1 fusion core. Total energy (**upper left**), RMSDs (**upper right**) of protein and RMSDs ligand (**lower left**).

**Figure 2:**
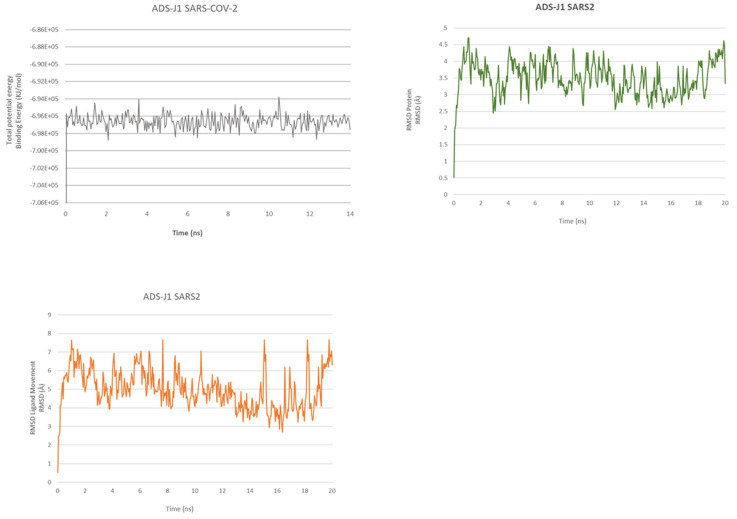
ADS-J1@SARS-CoV-2 fusion core. Total energy (**upper left**), RMSDs (**upper right**) of protein and RMSDs ligand (**lower left**).

We have also performed an MD of 20 ns for ADSJ with SARS-CoV-1 and SARS-CoV-2 to better analyze the interaction and stability of the compound (Figure 1 and Figure 2).

The MD simulation of 20 ns for ADS-J1 in complex with SARS-CoV-1 fusion confirms the stabilization of the molecule thanks to the high network of interactions. It shows that the binding energy for the overall structure stabilizes after 550 ps. After a slight energy increase at the start, the complex maintains a slight fluctuation, with less energy in the simulation from 8 ns to 10 ns. The ligand’s RMSD starts to stabilize after 1.35 ns, similar to the protein’s RMSD. After 20 ns of simulation, ADS-J1 still occupies the hydrophobic pocket with the phenyl group, but terminal groups have moved from their respective cavities.

The SARS-CoV-2 in the MD of ligand–protein complexes can spot a stabilization of the complex after 50 ps, but the ligand reaches stability after 650 ps. The ligand RMSD continues all the rest of the dynamic swinging from 3.8 Å to 7.2 Å, as with protein RMSD, which also stabilizes after 650 ps, and protein RMSD continues all the rest of the dynamics, swinging from 2.8 Å to 4.4 Å.

At the end of the MD, the ligand has slightly folded up but keeps interesting interaction as a hydrogen bond between the sulfonic acid and Lys947.

Overall, we can see fewer interactions and stability during the dynamic between HR1 of SARS-CoV-2 and ADS-J1 compared to SARS-CoV-1.

### 2.3. Peptides Docking and Analysis

Using the HDOCK server, we performed docking calculations for HR1 and HR2 interaction and the EK1 peptide interacting with SARS-CoV-1 before starting with the docking of multiple peptides of SARS-CoV-2. A first attempt was performed by docking the SARS-CoV-2 HR2 domain to the SARS-CoV-2 HR1 fusion core, providing a score of –58.72 kcal/mol.

All the generated poses from the server have been analyzed. We have selected and tabulated peptides in a helical conformation that targeted the fusion core site and bound to each interface of the trimeric HR1 bundle. Table 2 illustrates the results from the server, listing the mentioned docking tests at the top and the first 50 best-scored peptides.

### 2.4. Focus on the Interactions of the Best-Scoring Compounds

#### 2.4.1. NF 023 Hydrate

The first compound selected is identified as PubChem-24278597, known as NF 023 hydrate, an Adenosines/P2 nucleotide receptor antagonist.

The docking score for this compound with SARS-CoV-2 fusion core (PDB ID: 6LXT) is −12.03 kcal/mol (Table S1). NF 023 hydrate presents sulfonic acid groups linked to terminal naphthalene rings on each side, similar to ADS-J1, and a central hydrophobic portion, so, similar to the pharmacophore model, it creates a network of interactions.

The docking results showed hydrogen bonds involving all sulfonic acid groups and the central portion of the molecule. Donor residues are in the bottom part of the binding site with Ser 943 and Gln949 from helix B and C, respectively, interacting with the oxygen of two sulfonic acid groups (with a distance of 1.70 Å and an angle D-H-A of 169.6° for Ser943 and a distance of 1.82 Å and an angle of 158.0° for Gln949), as does Lys947 (1.72 Å with an angle of 160.2°) from helix B. On the upper side, other oxygen atoms of the naphthalene’s sulfonic acid groups create interactions with Gln926 (1.90 Å and 2.87 Å), Ser929 (1.64 Å with a D-H-A angle of 165.0°) and Gln935. One residue acceptor, Ser937 (B helix), also makes two bonds with an oxygen atom at a distance of 2.16 Å and 2.45 Å.

There are also attractive charge and *π*–cation interactions for Lys947 and Lys933 (Helix B), *π*–alkyl interactions for Ala942 and Leu938 (Helix C), plus Lys933, Ala944 and Lys947 (Helix B) with aromatic rings; carbon–hydrogen bonds for Gly946, Thr941 and Ser940 are also present. The most important interactions are salt bridges that, as for ADS-J1, stabilize the compound thanks to the terminal negatively charged sulfonic acid groups interacting with positively charged Lys933 (two bonds 1.89 Å and 2.15 Å) and Lys947 (1.77 Å).

The central part of the compound is placed in the central pocket involving interactions with Ser937, Leu938, Thr941 and Ala942.

**Figure 3:**
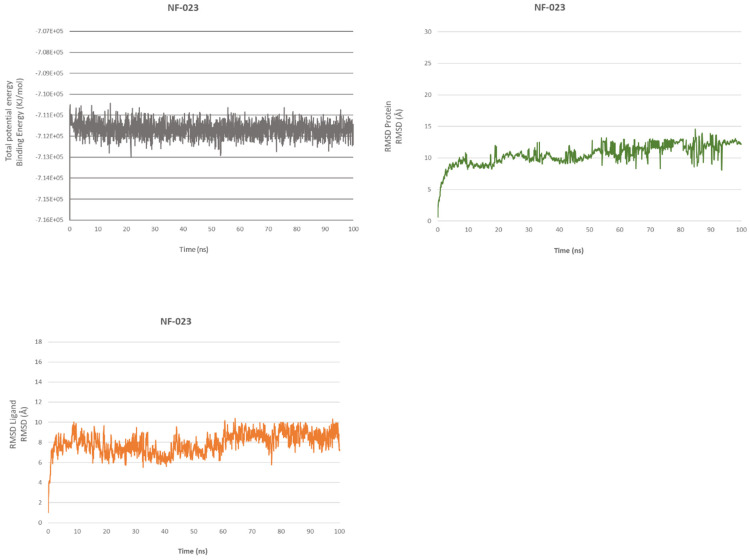
NF 023@HR1 domain. Total energy (**upper left**), RMSDs of protein (**upper right**) and RMSDs of ligand (**lower left**).

**Figure 4:**
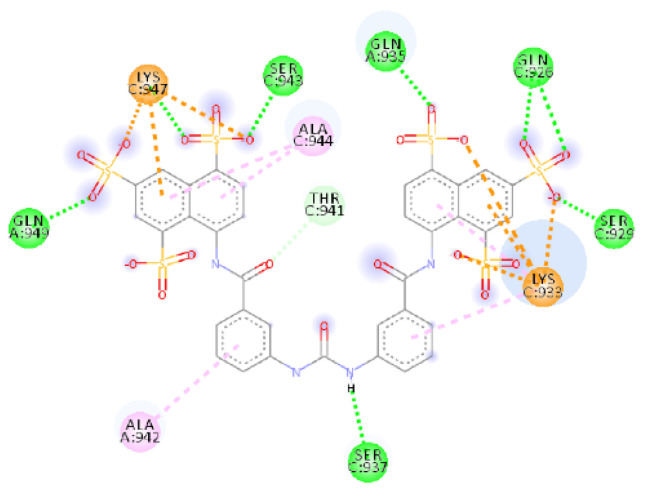
Calculated 2D interactions for NF 023 hydrate in complex with SARS-CoV-2’s HR1 domain.

MD confirms the high affinity with the protein; the simulation showed favorable binding energy for the overall structure that stabilizes after 50 ps and maintains a slight fluctuation of 956 kcal/mol regarding ADS-J1 with SARS-CoV-1, while RMSD for ligand and protein (Figure 3 and Figure 4) reaches equilibrium after 400 ps. After 20 ns simulation, NF 023 hydrate still occupies the hydrophobic pocket with the phenyl groups, and Ala944 (helix B), Leu945 and Ala942 (helix C) stabilize the central portion of the ligand through *π*–alkyl interactions. Lys947 still engages two *π*–cation interactions (4.50 Å and 4.66 Å) and a charge–charge interaction of 5.45 Å. Thanks to this interactions network, all the compound atoms are still along the surface of the fusion core.

#### 2.4.2. ZINC00097961973

The second selected compound is ZINC00097961973, which has two terminal aryl carboxylic acid groups on each side instead of the sulfonic groups and presents a central hydrophobic portion.

Our docking simulation found an affinity value of −10.73 kcal/mol (Table S1) for the best pose with the SARS-CoV-2 fusion core (PDB ID: 6LXT).

**Figure 5:**
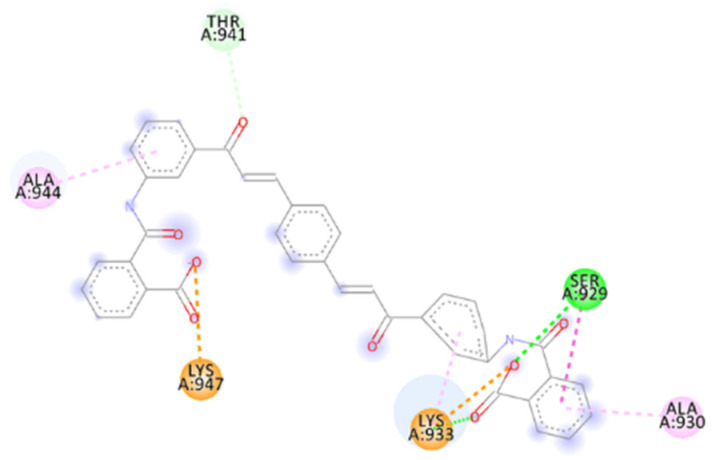
Calculated 2D interactions for ZINC00097961973 in complex with SARS-CoV-2’s HR1 domain.

**Figure 6:**
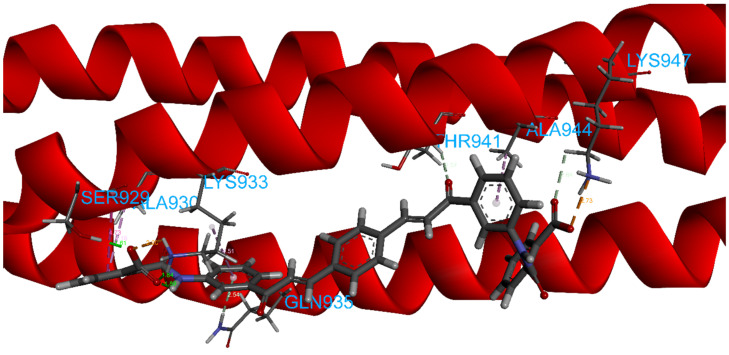
Details of calculated 3D interactions of ZINC00097961973 in complex with SARS-CoV-2’s HR1 domain.

The calculated 2D interactions were reported in Figure 5 and Figure 6. The molecule can engage two hydrogen bonds’ donor residues Ser929 (1.61Å with an angle of interaction of 171.7°) and Lys933 (1.84 Å with an angle of 151.5°) from helix B through its terminal carboxylic group.

Lys947 and Lys933 still play a critical role thanks to the cation–anion interaction, providing an attractive charge interaction (2.73 Å) and salt bridge (2.20 Å and a dihedral angle of −50.45°) fixing in the protein’s surface in the terminal parts of the ligand.

Other reported interactions are carbon–hydrogen bonds between Lys947 and Thr941 with the oxygen of the terminal carboxylic acid and the other oxygen of the internal carboxyl group, a hydrogen-bond donor interaction involving Gln935 (2.54 Å), an amide–*π*-stacked interaction with Ser929 (4.73 Å) and a *π*–alkyl interaction for Ala944 and Ala930.

MD simulation shows an overall stabilization for the complex over time. From the starting point at the fixed docking pose (time 0), the complex takes 500 ps to reach energy-binding equilibrium. The RMSDs of the ligand and the protein stabilize after 1 ns, with the ligand fluctuating from 4 Å to 6 Å, but it mostly keeps its value between 4 Å and 5 Å, and the protein keeps its value around 4–5 Å (Figure S1).

In all three faces of the domains, the compound still occupies the hydrophobic pocket and maintains different interactions. Among all, there are hydrogen bonds between Gln949 and carboxylic acid (2.44 Å), Gln935 that binds with hydrogen from the nitrogen of the amide as an acceptor (distance 1.83 Å, D-H-A angle 155.9°) and with the carboxylic acid as a donor (1.89 Å, angle 176.3°) and Lys933 (2.35 Å), which also forms *π*–cation and attractive charge interactions. There are also other *π*–alkyl interactions.

Moreover, the MD of 20 ns seems to confirm the stability of the complex and RMSDs.

Taking advantage of these promising results, we performed a new 100 ns MD simulation involving the whole spike protein in a post-fusion conformation (PDB ID: 6XRA) [15].

Even considering the entire structure of the spike protein, we can see a stabilization of the total energy for the complex after 0.3 ns. It maintains stability for the rest of the dynamic simulation, with a minimum value for the binding energy of −170,291 kcal/mol and −169,694 kcal/mol as the highest energy. The RMSD of the ligand stabilizes after 1.5 ns, and there are no essential fluctuations for the rest of the MD. While the protein RMSD reaches equilibrium after 2 ns, we see some fluctuations due to the protein changes in conformation. Even during the 100 ns MD, the HB5D interaction shows good stability.

#### 2.4.3. ZINC000150368097

The third selected compound from our calculation was ZINC000150368097. This molecule is structurally similar to ADS-J1, containing sulfonic acid groups and diazonium bridges. The docking calculation resulted in a binding affinity of −10.68 kcal/mol for the selected pose (Table S1).

**Figure 7:**
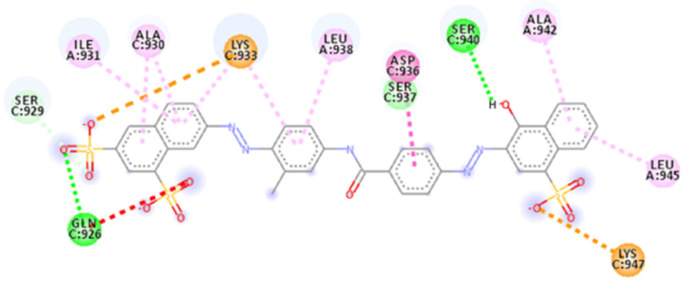
Calculated 2D interactions of ZINC000150368097 in complex with SARS-CoV-2’s HR1 domain.

Calculated 2D interactions were reported in Figure 7. For this molecule, two hydrogen bonds are identified with Gln926 as a donor residue interacting with sulfonic acid (2.20 Å) and Ser940 as an H-bond acceptor interacting with a hydroxylic group (1.85 Å), both from helix B.

Terminal portions containing sulfonic acids are involved in attractive charge interaction with the key residues Lys933 (4.32 Å) and Lys947 (5.15 Å). Moreover, Lys933 is also engaged in a *π*–alkyl interaction.

Other interactions include van der Waals, carbon–hydrogen bond and *π*–alkyl with Ser929, Ala930 from helix B and Ile931, Leu938, Ala942 and Leu945 from helix C. There is also an amide–π-stacked interaction for Asp936 (4.92 Å).

MD simulation shows a stabilization for the binding energy of the complex after 100 ps. However, the RMSDs of the ligand and protein do not maintain a stable equilibrium during the simulation. The ligand RMSD during the simulation rises from 5 Å to 15 Å from 150 ps to 8 ns (Figure S2). It then decreases and reaches stability between 10 and 18 ns. The protein RMSD follows a similar pattern.

The compound remains well packed to HR1 and maintains several interactions, including H-bonds with Lys933 (2.28 Å, angle 146.6°) and Ser940 (2.06 Å). Moreover, there are other interactions, such as charge–charge interaction between Lys 947 and the sulfonic acid (4.20 Å) as it occurs with Lys933 in the upper side (5.04 Å), and *π*–cation and *π*–alkyl interactions that stabilize the complex.

#### 2.4.4. ZINC000097996131

Another selected molecule is ZINC000097996131, which contains different aryl groups and terminal carboxylic acids. The compound is registered as PubChem-3098477 and deposited for use as a heparanase inhibitor.

**Figure 8:**
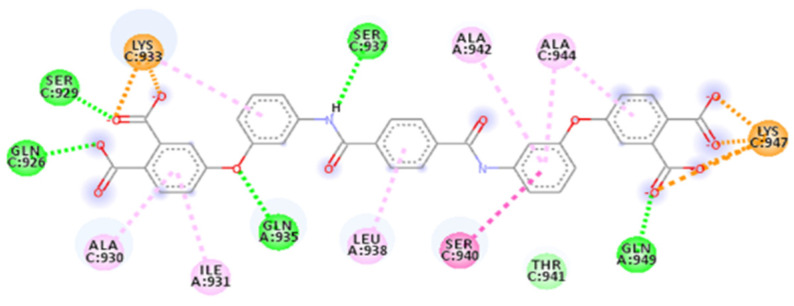
Calculated 2D interactions of ZINC000097996131 in complex with SARS-CoV-2’s HR1 domain.

The calculated docking score is −10.38 kcal/mol (Table S1). The calculated 2D interactions were reported in Figure 8. The results are validated by a dense network of interactions, among which are seven H-bonds involving, from helix B, residues such as Gln926, which makes two hydrogen bonds with carboxylic acid (1.80 Å, with an angle of 157.9° and 2.52 Å). Moreover, interactions of Ser929 and Lys933 with another carboxylic acid (1.56 Å, angle 154.2° and 1.80 Å each) are present, and Ser937 acts as an H-bond acceptor with the amide group (distance 1.95 Å, with an angle of the interaction of 152.5°). In helix C, there are Gln949 and Gln935 as H-bond donors, creating interactions with a terminal carboxylic acid on the bottom side and oxygen bridge (1.74 Å, angle 169.1° and 1.96 Å, angle 166.7°). There are then interactions for Lys933, making a salt bridge with a negatively charged carboxylic acid in the upper zone (1.86 Å), and Lys947, which makes two salt bridges with the other side with the carboxylic acid (1.72 Å and 1.75 Å). Among others are van der Waals, *π*–alkyl and amide–π-stacked interactions for Lys933, Ala942, Ala944, Ser940, Leu938, Ile931 and Ala930.

Analyzing the 20 ns MD simulation, we can see that the complex reaches equilibrium after 100 ps. The highest variation in energy is around 1673 kcal/mol, but the energy is overall stable during the simulation. The ligand and protein RMSDs rise to 650 ps, then decrease and finally increase again to stabilize around 6 Å for the ligand and 4 Å for the protein (Figure S3). The compound remains stable in the fusion core, positioning its hydrophobic groups in the central pocket.

Ser937 still acts as an H-bond acceptor with amide (2.05 Å interaction); moreover, Gln935 makes two hydrogen bonds with a carboxylic acid in the upper side (1.70 Å) and with amide oxygen (1.70 Å). Lys933 plays an essential role in making *π*–cation interaction (4.51 Å) and amide–*π*-stacked interaction (4.61 Å). Finally, there are other interactions, such as attractive charge (Lys947 4.94 Å), *π*–donor H-bond and *π*–alkyl.

#### 2.4.5. PubChem-66982178

Compound PubChem-66982178 showed an affinity of −10.12 kcal/mol (Table S1) for the fusion core. It has been patented as a Hepatitis C virus inhibitor (EP-2499127-B1).

This compound has different aryl groups, heterocycle and terminal carbamic groups.

**Figure 9:**
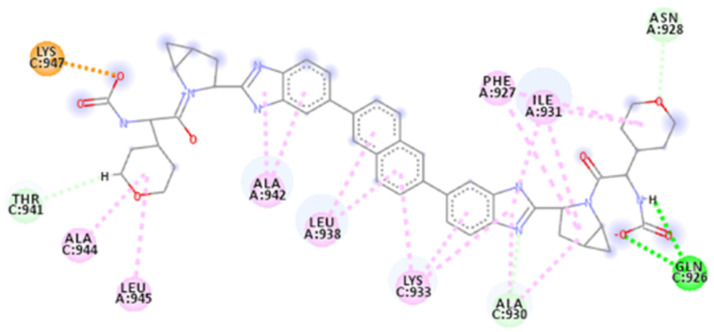
Calculated 2D interactions of PubChem-66982178 in complex with SARS-CoV-2’s HR1 domain.

Calculated 2D interactions were reported in Figure 9. The ligand located itself along the face of the two helixes, with the upper rings between Gln926 and Asn928; benzimidazole group allocates in a pocket between Lys933 and Gln935, naphthalene interacts with hydrophobic pocket and the inferior side folds up, placing it in the right side of the face.

Gln926 is the only residue that makes hydrogen bonds. It includes H-bond donor interaction with a carbamic group (2.05 Å, D-H-A angle 143.5°) and acts as an H-bond acceptor, interacting with the hydrogen of the nitrogen from the carbamide group (2.00 Å, angle 142.4°).

Lys947 makes a salt bridge with the carbamide (2.07 Å), establishing a carbon–hydrogen bond and alkyl and *π*–alkyl interactions with Asn928, Ile931, Ala944, Leu945, Leu942, Leu938, Lys933 Ala930 and Phe927 to stabilize the pose of the compound in the fusion core.

MD calculations confirm the overall stability of the complex that, after 200 ps, reaches equilibrium. The protein and ligand RMSDs achieve stability after 200 ps and 250 ps each, with the protein having an average distance of 5.5 Å and the ligand maintaining its equilibrium at around 6 Å (Figure S4). The central hydrophobic portion of the ligand, from the beginning of the MD to the end of the simulation, maintains its position near the central hydrophobic pocket of the protein. At the same time, the bottom side folds up, although it places itself on the left side of the trimer’s face. Lys933 makes an attractive charge interaction with the terminal amide in the upper zone of the binding site (4.69 Å); meanwhile, there are other alkyl and *π*–alkyl interactions with Lys933, Ala930, Ala944 and Leu945, and van der Waals interactions with Ser939 and Ala942.

#### 2.4.6. AP00094

Among our best-resulting peptides, we have chosen AP00094 from the APD3 database (https://wangapd3.com/database/query_output.php?ID=00094, accessed on 1 June 2022). This peptide, named Temporin A, has a 13 residues sequence and has been isolated from the European common frog. Temporin A has several functions; among these, it is anti-Gram-positive and Gram-negative. Its biological activity has been confirmed with different approaches, such as structural analysis, northern blot analysis and antimicrobial assay. It has also shown antiviral, antifungal, candidacidal, antiparasitic, chemotactic, wound healing and anticancer activity, exhibiting selective cytotoxicity against lung cancer cells [45,46,47].

**Figure 10:**
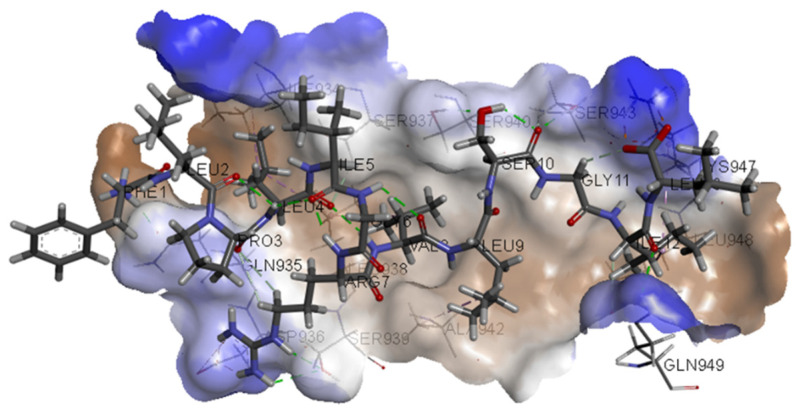
Calculated 3D interactions of AP00094 in complex with SARS-CoV-2’s HR1 domain.

The docking score obtained for AP00094 in the HDOCK server is −196.37 (Table 2). It has different interactions and occupies the fusion core, including the hydrophobic pocket (Figure 10).

The set of calculated interactions involves H-bond acceptor residue, such as Gln935, which makes two interactions (1.82 Å and 1.97 Å), and H-Bond donor residue, such as Ser940 (1.88 Å), Ser943 (1.85 Å) and Gln949 (2.15 Å). There are salt bridges interactions for Asp936 (which makes two bridges, 1.76 Å and 2.08 Å) and Lys947 (which also makes two more bridges, 1.78 Å and 1.81 Å).

Further, *π*–alkyl interaction, carbon–hydrogen bond and van der Waals interactions involving residues such as Lys933 (4.92 Å *π*–alkyl interaction), Asp936, Ser937, Leu938, Ser940, Ala942, Lys947, Leu948 and Gln949 are also present. Furthermore, the peptide stabilizes its interactions thanks to intramolecular interactions (hydrogen bond and *π*–alkyl interaction).

The MD simulation of 20 ns shows general stability. In fact, the complex reaches good equilibrium after 100 ps and keeps it for the rest of the MD simulation. The protein needs 150 ps to stabilize, then maintains a good value range while the ligand reaches stability after 400 ps (Figure S5).

The peptide, during the simulation, keeps its location near the fusion core, and it is taking interaction with the hydrophobic pocket; it still shows two hydrogen bond interactions with Gln935 (2.09 Å and 2.95 Å), Ser937 (2.12 Å), Lys947 (1.67 Å, 2.50 Å and 2.54 Å) and Gln949 (2.00 Å). There is an attractive charge interaction between Asp936 and the peptide’s arginine (4.38 Å). Then, the peptide keeps stabilizing itself thanks to other interactions, such as *π*–alkyl interaction, carbon–hydrogen bond and van der Waals interactions. Finally, Asp936 keeps a charge–charge interaction of 4.38 Å.

#### 2.4.7. AVP1227

AVP1227, also called GBVA5, is a peptide selected from the AVPdb database (http://crdd.osdd.net/servers/avpdb/, accessed on 1 June 2022). It is a 17 amino acids compound derived from GBVA non-structural protein 5A used against HCV.

To identify new HCV inhibitors, several peptides from C5A-corresponding NS5 protein have been synthesized. Further optimization of helicity and hydrophobicity provided good improvements, leading to potent inhibitory activity [48].

The docking score for this peptide on the HDOCK server is −191.20 (Table 2).

Interactions for this peptide include hydrogen bond and charge interactions with Lys933 and Lys947, which play an essential role in anchoring the inferior part of the peptide.

Between H-bond donors, there are Gln926 (2.15 Å and 1.98 Å), Asn928 (1.82 Å), Ser929 (1.72 Å), Lys933 (1.80 Å), Gln935 (2.00 Å and 1.94 Å), Gln949 (1.85 Å and 1.76 Å) and Lys947 (1.78 Å), while Gln926 (2.15 Å) and Ser943 (2.01 Å) residues act as an H-bond acceptor.

Salt bridge interactions involve Lys933 with a Glu residue (1.82 Å), while there is a salt bridge interaction between Lys947 and another Glu residue from the peptide (2.72 Å). Further interactions, such as *π*–alkyl, carbon–hydrogen bond and van der Waals, occur.

For this peptide, an MD of 20 ns was performed. After the simulation, the peptide maintains the interactions within the pocket.

The graphics (Figure S6) show us that AVP1227 reaches overall equilibrium after 100 ps; the protein and ligand are also stabilized after 250 ps and 150 ps, respectively, with values around 3 Å for the protein and 4 Å for the ligand. The peptide at the end of the dynamic simulation maintains some H-bond interaction, such as the one with Gln935 (1.89 Å) and Ser940 (2.48 Å), the salt bridges with Asp936 (1.76 Å and 1.84), a charge interaction and other interactions, such as carbon–hydrogen bonds, *π*–alkyl interactions for Lys933, Leu938, Ale942 and Leu945.

#### 2.4.8. Salvianolic Acid C

Among the natural compounds, Salvianolic acid C (Sal-C) was selected thanks to its good fusion inhibition activity toward the SARS-CoV-2 6-HB domain [49].

This study used docking analysis to target the fusion core to identify the binding site. The results show an affinity of −7.60 kcal/mol and an IC_50_ of 1.71 μM, with a low affinity for SARS-CoV-2 RBD.

We then performed a re-docking analysis and MD simulation for Sal-C to test the compound validity and compare the founded interactions with the well-known HR1–HR2 complex interactions in SARS-CoV-2.

The result shows a good docking score to confirm the good activity of Sal-C as a fusion inhibitor. This compound is smaller than ADS-J1 and seems quite different but shows some important relationship with it. In fact, it also has a terminal acid that, in this case, is a carboxylic one, while, on the other side, there are two hydroxylic groups. The central part of the molecule contains one unsaturation and a benzofuran ring.

**Figure 11:**
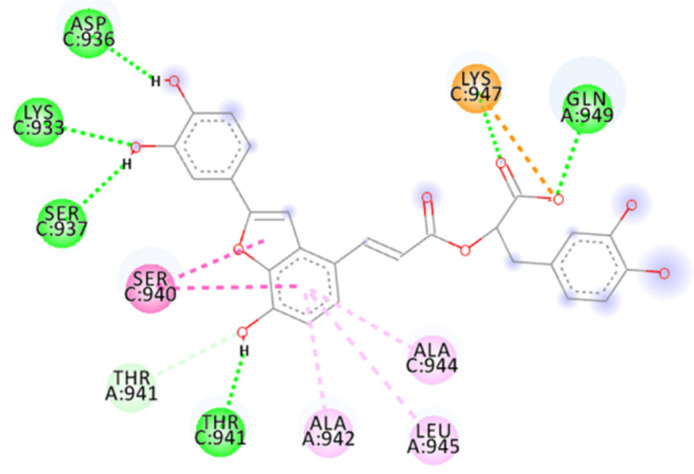
Calculated 2D interactions of Sal C in complex with SARS-CoV-2’s HR1 domain.

**Figure 12:**
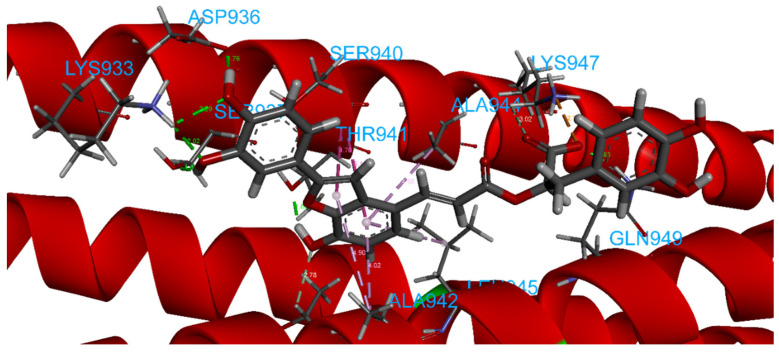
Details of calculated 3D interactions of Sal C in complex with SARS-CoV-2’s HR1 domain.

The calculated docking binding energy was −8.75 kcal/mol (Table S1), confirming the importance of interactions with Lys933 and Lys947 and especially the importance of the occupation of the hydrophobic pocket (Figure 11, Figure 12 and Figure S10).

The hydrogen bond interactions involve Gln949 as a donor residue, which interacts with carboxylic acid (1.89 Å, and an angle D-H-A of 162.6°), a Lys933 that makes two hydrogen bonds with two oxygen atoms from hydroxylic groups (2.29 Å and 2.02 Å), Asp936 as an acceptor residue that binds with hydrogen from the same hydroxylic groups (1.76 Å, angle 154.5°), Ser937 (1.83 Å, angle 148.2°) and Thr941 (1.83 Å), as well as many more interactions, such as carbon–hydrogen bonds involving Lys947, Thr941 or amide–π-stacked for Ser940 with benzofuran (two interactions 4.76 Å and 5.13 Å). The benzofuran at the central portion is important for establishing a hydrophobic interaction with the pocket in the central portion of the fusion core; it involves *π*–alkyl interaction with Ala942, Ala944 and Leu945. Lys947 represents a key residue providing an attractive charge interaction with a carboxylic acid (2.78 Å), fixing the compound’s position.

The binding energy of the complex stabilizes after 0.2 ns of the MD simulation and maintains overall stability for the rest of 20 ns MD.

The RMSDs of the ligand and protein have similar graphs; they stabilize around 1.5 ns but then maintain a slight increase in values ranging from 10.50 Å to 14.50 Å for the ligand and from 4.50 Å to 5.50 Å with a maximum value of 6 Å for the protein (Figure S7).

#### 2.4.9. ZINC000150346512

Not much is known regarding the activity of this compound; bioactivity prediction with SwissTargetPrediction and ChemBL shows possible interaction with Lysophosphatidic acid receptor 5 (LPAR5). This ligand is more structurally similar to ADS-J1 as it contains a terminal aryl ring on each side, with two carboxylic acids per. It also contains three other rings, with the central one linked to a phthalimide.

This compound presents a high number of interactions, including hydrogen bonds with various donor residues, such as Gln935, which interacts with an oxygen bridge (1.95 Å, D-H-A angle of 164.5°) from helix A and helix C. Gln926 makes a hydrogen bond with carboxylic acid (1.75 Å, angle 172.8°), as well as Ser929 (1.53 Å, angle 173.9°). Ser937, an acceptor of the hydrogen bond, interacts with the hydrogen of the amide group (1.95 Å, angle 148.4°). On the underside, carboxylic acid involves Ser943 (1.62 Å, angle 177.0°) and Lys947 (1.74 Å, angle 155.0°). Gln949, from helix A, binds to the oxygen of carboxylic acid (two hydrogen bonds 1.86 Å, angle 153.3° and 2.85 Å). Finally, phthalimide binds with its carboxyl to Ser940 (1.80 Å).

Two central aryl rings occupy the hydrophobic pocket and are further stabilized thanks to other interactions, such as carbon–hydrogen bonds and *π*–anion and *π*–alkyl involving Ala930, Lys933, Ala944 and Asp936.

Lastly, an important role is played by Lys 933 and Lys947. These two residues are reconfirmed as an anchor for terminal groups of the compound, stabilizing, even more, the binding pose of the molecule. Lys933 makes two salt bridges of 1.75 Å and 1.79 Å with carboxylic acid, while Lys947 makes two attractive charge interactions (2.91 Å and 3.51 Å).

We performed an MD simulation of 20 ns for this compound. The resulting binding energy stabilizes after 0.25 ns. Afterward, the complex reaches good equilibrium, with an average value of −50.4 kcal/mol. The RMSD for the ligand and protein have the same trend: they stabilize after 0.5 ns and have only some light fluctuations. Their values during the simulation are around 5 Å for the ligand and 4.2 Å for the protein.

#### 2.4.10. Marine_160925_88_2

Another interesting natural compound was marine_160925_88_2. *SwissTargetPrediction* suggests for this compound a possible interaction with Caspase 3 and 7. It has a terminal side with a carboxylic acid and a pyranose group on the other side that remembers the aryl ring with hydroxyl groups of Sal C. The central hydrophobic portion contains unsaturation and another ring.

The in silico analysis has found an affinity of −10.21 kcal/mol for the natural compound (Table S1).

**Figure 13:**
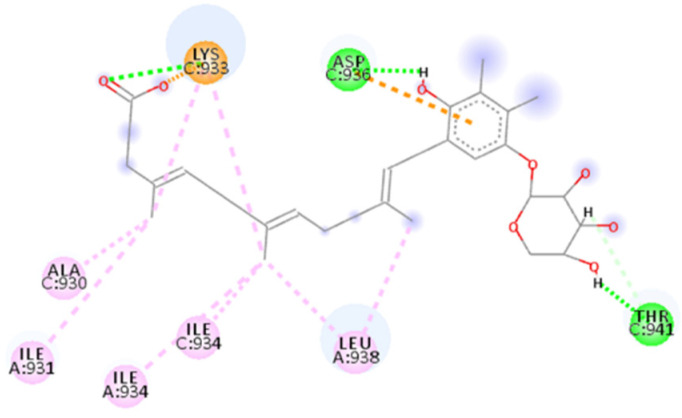
Calculated 2D interactions of marine_160925_88_2 in complex with SARS-CoV-2’s HR1 domain.

Calculated 2D interactions were reported in Figure 13. The molecule shows a high network of bonds, such as conventional hydrogen bond interaction of carboxylic acid with Lys933 as hydrogen bond donor (1.95 Å), while Asp936 interacts with a hydroxylic group from the aryl ring in the central portion of its structure. Finally, Thr941 and the sugar ring act as hydrogen bond acceptors (2.05 Å, D-H-A angle 167.5° and 1.95 Å, angle 178.8°, respectively).

Ser940 and Thr941, from helix B and C, respectively, are involved in carbon–hydrogen bonds. Moreover, several alkyl interactions involving Lys933 make four interactions: Ala930 and Ile934, from helix B, with the residues Ile931, Ile934 and Leu938, from helix C, each one creating two interactions. There is also a *π*–anion interaction for Asp936. Lys933, as usual, also makes a salt bridge (2.51 Å) with a terminal carboxylic acid. In the generated pose, the glycosidic ring and alkenyl chain occupy the fusion core’s hydrophobic pocket.

The MD simulation of 20 ns shows overall stability for the complex, stabilizing its binding energy after 0.3 ns and maintaining a good trend for all the simulations in the range of −57.54 kcal/mol (Figure S8). In the RMSD of the ligand, we can see that it reaches good equilibrium after 1 ns, with an average value of 7 Å, while the protein RMSD shows some fluctuations in 8–10 ns intervals, while the remaining RMSD is around 3.5 Å.

#### 2.4.11. Thalassiolin A

Thalassiolin A showed good activity, with a docking score of −9.94 kcal/mol (Table S1). This natural compound is isolated from a marine plant, the Caribbean seagrass *Thalassia testudinum*, with two other substances, Thalassiolin B and C. These small molecules are characterized by substituting a sulfated *β*-d-glucose at the 7-position.

One important use for Thalassiolin A is as an inhibitor of HIV cDNA integrase, tested in vitro and in silico, indicating the catalytic core as a binding spot [50]. Moreover, Thalassiolin B reduces skin UVB-induced damage [51].

**Figure 14:**
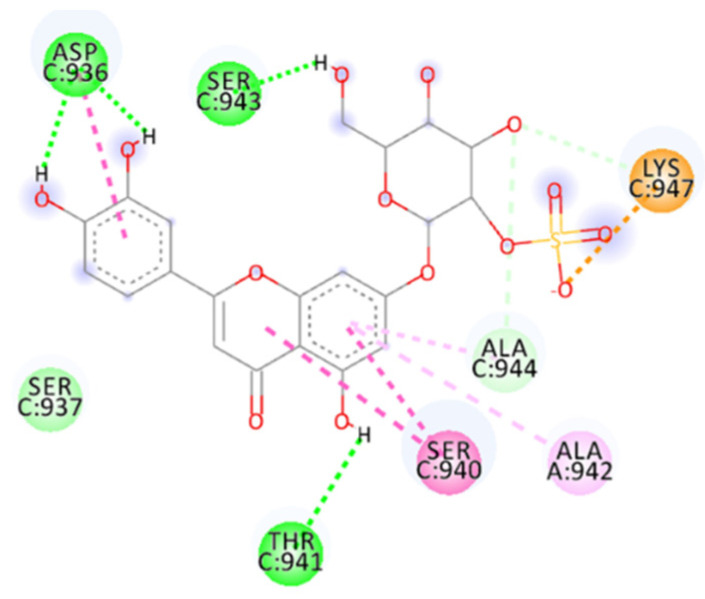
Calculated 2D interactions of Thalassiolin A in complex with SARS-CoV-2’s HR1 domain.

The calculated 2D interactions were reported in Figure 14. This compound contains a terminal anionic group, the sulfonic acid group, that is crucial for interacting with the fusion core and responsible for forming a salt bridge of 1.77 Å with the positively charged Lys947.

A high number of other interactions stabilize the ligand even more. The hydrogen bonds involve hydrogen bond acceptor residues of Asp936, which binds to two terminal hydroxylic groups (two bonds of 1.64 Å, D-H-A angle 155.9 Å and 1.70 Å angle 170.8°), Thr941 with a central hydroxylic group (2.75 Å) and Ser943 with a hydroxylic group from the pyranose ring (1.78 Å). Other interactions are represented by van der Waals forces, carbon–hydrogen bonds and pi–alkyl interactions; these bonds involve Als942 and Ala944 residues. Finally, there are amide–pi stacked interactions for Asp936 (4.52 Å) and Ser940 (which form two interactions of 4.09 Å and 4.83 Å).

The central portion of the compound, containing fused rings, as with other interesting molecules, shows good interaction, positioning itself in the hydrophobic pocket between Lys933 and Lys947.

The binding affinity of the complex stabilizes after 0.2 ns and maintains good stability for the rest of the MD simulation (Figure S9).

#### 2.4.12. SN00114935

Another natural compound that showed good predicted activity is SN00114935 (PubChem-10169258), with a docking score of −8.26 kcal/mol (Table S1).

This compound counts two aryl rings on each side with carboxylic acid substituents; an ester bridge links both rings to the central body made of four rings of six atoms and one ring of five atoms fused.

**Figure 15:**
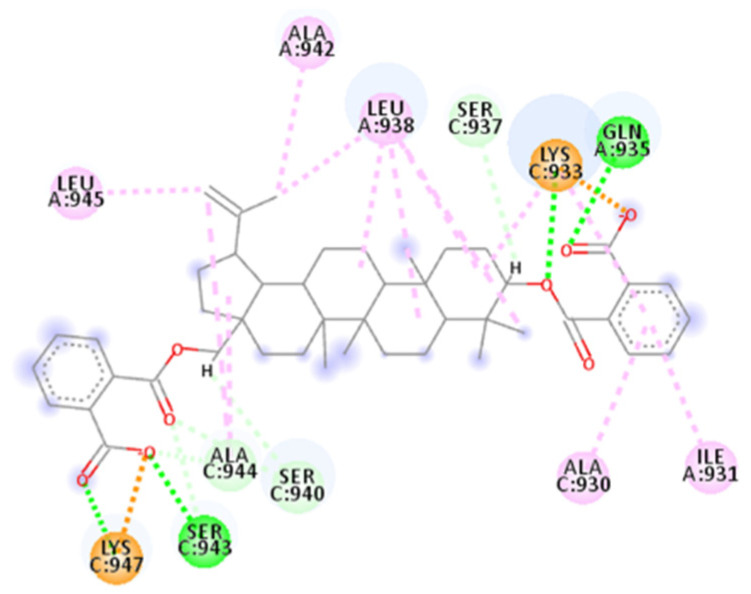
Calculated 2D interactions of SN00114935 in complex with SARS-CoV-2’s HR1 domain.

The calculated 2D interactions were reported in Figure 15. This molecule has an important network of interaction involving different residues, among which hydrogen bond donors, such as Gln935 from helix A, make a bond with carboxylic acid (1.82 Å, angle 165.1°), and from helix C there are Lys933 binding to the oxygen of ester (2.02 Å, angle 159.0°), Ser943 and Ala944 both binding to the carboxyl of ester (one with a distance of 1.78 Å, an angle of 167.7° and the other residue 2.77 Å) and Lys947 (1.79 Å).

The other interactions are carbon–hydrogen bonds for Ser940 and Ser943, pi–alkyl interactions for Ala930 (helix C), Ile931 (helix A), Lys933 (helix C) and alkyl interactions with fused rings and the propenyl group for Lys933, Leu938 (this residue makes five alkyl interactions), Ala942, Leu945 and Ala944.

As usual, an important role is played by Lys933, which makes a salt bridge of 1.72 Å, and Lys947, which also makes one salt bridge of 1.85 Å; both bridges involve terminal carboxylic acid groups.

The central portion with five fused rings mainly occupies the hydrophobic pocket. MD analysis shows binding energy for the complex that stabilizes after 0.1 ns.

While the RMSDs of the protein and the ligand stabilize after 350 ps, there is a rising trend for the ligand, with a minimum ranging around 4 Å at the start and ending near 8 Å. Moreover, the protein seems stable enough, with an average value of 4.5 Å during the 20 ns of MD.

## 3. Methods and Materials

### 3.1. PBVS and Selected Databases

The pharmacophore model was built using Pharmit by inserting the HR1 domain of SARS-CoV-2’s post-fusion core of the S2 subunit (PDB ID: 6LXT) and the HR2 domain of the same PDB as input. Pharmit parameters for 3D-pharmacophore research have been modified for an optimal search. The spheres considered involved hydrogen bond donor interactions for a residue of Ser1196 with Ser929, hydrophobic interactions for Leu1193 with the HR1 pocket (involving Ile931), hydrogen bond acceptor interactions for Asp1194 (interacting with Gln935 and Gln1180 with Gln949) and, finally, Glu1182 and Glu1195 charge–charge interactions with Lys947 and Lys933, respectively. The radius of each of the selected spheres has been increased to a less selective 1.5 Å for H-bonds and 1 Å for hydrophobic and charge interactions.

This model was used for the virtual screening of FDA-approved drugs, marine natural products (MNP), MolPort, ZINC, ChemSpace, PubChem, CHEMBL25 and supernatural products II Marvin (MNP) for a total of more than 166149376 molecules and 960875860 conformers. The search focused on selecting one only orientation for each conformation of the molecules. The resulting compounds have been further minimized according to the functions of Pharmit.

### 3.2. Structure Preparation and Minimization

The structures of all the molecules used in this study were built using Marvin Sketch (18.24, ChemAxon Ltd., Budapest, Hungary, http://www.chemaxon.com, accessed on 1 June 2022). A first molecular mechanics energy minimization was used for 3D structures created from the SMILES; the Merck molecular force field (MMFF94) present in Marvin Sketch was used. The protonation states were calculated, assuming a neutral pH. The PM3 Hamiltonian, implemented in the MOPAC package (MOPAC2016 v. 18.151, Stewart Computational Chemistry, Colorado Springs, CO, USA) [52], was then used to further optimize the 3D structures before the alignment for the docking calculations.

### 3.3. Peptides Docking through HDOCK Server

The HDOCK server was chosen as a tool for modeling and docking protein–protein complexes. Overall, the HDOCK server obtained significantly better performance in binding mode prediction than template-based modeling and traditional free unbound docking for protein–protein docking [53]. The server was used (http://hdock.phys.hust.edu.cn/, accessed on 1 June 2022) to analyze up to 400 small peptides ranging from 12 to 20 amino acids. They were selected for their antiviral activity based on virus-entry inhibition. The servers used for the research of peptides’ sequence are AVPD3 (https://wangapd3.com/main.php, accessed on 1 June 2022) and AVPdb (http://crdd.osdd.net/servers/avpdb/, accessed on 1 June 2022), which offer an advanced research tool and various information about each peptide. We have uploaded the receptor PDB file (the entire structure of the SARS-CoV-2 spike protein in post-fusion conformation, PDB ID: 6LXT) and used sequence submission for the peptide. All the other settings were left to the default setting.

### 3.4. Molecular Docking

Flexible ligand docking experiments were performed by employing AutoDock 4.2.6 and AutoDock Vina software implemented in YASARA (v. 19.5.5, YASARA Biosciences GmbH, Vienna, Austria) [54,55] using the three-dimensional crystal structures of the fusion core of SARS-CoV-1’s spike protein (PDB ID: 1WNC) [56], the post-fusion core of S2 subunit (PDB ID: 6LXT) and the full structure of the SARS-CoV-2 spike protein in post-fusion conformation (PDB ID: 6XRA) obtained from the Protein Data Bank (PDB, http://www.rcsb.org/pdb, accessed on 1 June 2022) and the Lamarckian genetic algorithm (LGA). The proteins have been protonated and optimized using YASARA software. The maps were generated by AutoGrid (4.2.6) with a spacing of 0.375 Å and dimensions encompassing all atoms extending 5 Å from the surface of the structure of the crystallized ligand. Point charges were initially assigned according to the AMBER03 force field and then damped to mimic the less polar Gasteiger charges used to optimize the AutoDock scoring function. All parameters were inserted at their default settings, as previously reported. In the docking tab, the macromolecule and ligand were selected, and LGA parameters were set as ga_runs = 100, ga_pop_size = 150, ga_num_evals = 25,000,000, ga_num_generations = 27,000, ga_elitism = 1, ga_mutation_rate = 0.02, ga_crossover_rate = 0.8, ga_crossover_mode = two points, ga_cauchy_alpha = 0.0, ga_cauchy_beta = 1.0, number of generations for picking worst individual = 10.

### 3.5. Molecular Dynamics Simulations

The MD simulations of the complexes were performed with the YASARA structure package. A periodic simulation cell with boundaries extending 8 Å [57] from the surface of the complex was employed. The box was filled with water, with a maximum sum of all water bumps of 1.0 Å and a density of 0.997 g/mL.

The setup included optimizing the hydrogen bonding network [58] to increase the solute stability and a p*K*_a_ prediction to fine-tune the protonation states of protein residues at the chosen pH of 7.4 [59]. NaCl ions were added with a physiological concentration of 0.9%, with an excess of either Na or Cl to neutralize the cell. Water molecules were deleted to readjust the solvent density to 0.997 g/mL. The final system dimensions were approximately 80 × 80 × 130 Å^3^ for SARS-CoV-1 and SARS-CoV-2 protein/ligand complexes.

The simulation was run using the ff14SB force field [60] for the solute, GAFF2 [61], AM1BCC [62] for ligands and TIP3P for water. The cutoff was 10 Å for Van der Waals forces (the default used by AMBER) [63], and no cutoff was applied to electrostatic forces (using the Particle Mesh Ewald algorithm) [64]. The equations of motions were integrated with multiple time steps of 2.5 fs for bonded interactions and 5.0 fs for nonbonded interactions at a temperature of 298 K and a pressure of 1 atm (NPT ensemble) using algorithms described in detail previously [65,66]. Short MD simulation was run on the solvent only to remove clashes. The entire system was then energy-minimized using a steepest descent minimization to remove conformational stress, followed by a simulated annealing minimization until convergence (<0.01 kcal/mol Å). Finally, 10 ns MD simulations without any restrictions were conducted, and the conformations of each system were recorded every 200 ps. After inspecting the solute RMSD as a function of simulation time, the last 3 ns averaged structures were considered for further analysis.

## 4. Conclusions

The purpose of this article was to virtually screen a dataset of compounds in order to find molecules with high binding energies with the S2 domain of SARS-CoV-2 as it is the most conserved between the fusion proteins and has a primary role in the fusion process of the virus. We used the post-fusion conformational state of the S2 subunit and focused our docking simulation on the HR1–HR2 fusion core region as the target site for the study of the small molecules. We employed the HDOCK server for peptide docking using the same crystal structure with the no-site specification.

The validation of our procedure was performed by finding ADS-J1 with our methodology as an inhibitor of the fusion process. Interestingly, this compound was already reported and tested in silico and in vitro with good inhibitory activity against SARS-CoV-1 and HIV.

Our methodology was then applied to different databases of small molecules and new peptides. After the re-docking of ADS-J1 and the fusion core of SARS-CoV-1, we could confirm the activity of this compound with a docking score of −9.76 kcal/mol with our methodology. We then applied the same screening methodology for different databases. The most interesting compounds showed similar structural properties with a negative terminal charge at a physiological pH and a central hydrophobic portion. This seems to validate the key role of the hydrophobic pocket for HR1–HR2 interaction and for the inhibitor compound and the importance of Lys933, Lys947 and other residues, which grant stabilization for the pose thanks to the H-bond and charge–charge interactions.

Among these, NF 023 hydrate (MolPort-006-822-583) is one of the best-scored compounds, with an affinity of −12.03 kcal/mol. NF 023 hydrate showed essential interactions with the central hydrophobic portion and good stabilization thanks to multiple hydrogen bonds, charge interactions and other interactions. The MD simulation confirmed the validity of this compound. Moreover, ZINC00097961973, which has a central hydrophobic portion and presents two terminal aryl carboxylic acid groups on each side, showed an affinity of −10.73 kcal/mol. This compound has an extensive network of interactions, confirmed by a 100 ns MD using whole post-fusion spike protein.

Considering the compounds retrieved from natural sources, our screening led to the selection of several active compounds, among them Salvianolic acid, recently reported for its fusion inhibition activity [49], validating the reliability of our methodology. We also found Thalassiolin A with a docking score of −9.94 kcal/mol and marine_160925_88_2 with an interesting docking score of −10.21 kcal/mol as interesting compounds from natural sources.

The HDOCK server was used to identify novel peptide sequences. The results of this search were then validated with docking experiments and MD simulations. Two interesting active peptides were identified: AP00094 (Temporin A), isolated from the European common frog, and AVP1227 (GBVA5), already reported in the literature as an HCV inhibitor.

In conclusion, we can assume that the inhibition of the six-helix bundle core of SARS-CoV-2 is a valid target to inhibit virus entry. The discussed compounds reported in this paper encouraged future results for in vitro tests against SARS-CoV-2.

## Figures and Tables

**Table 1:** Screened databases, cutoffs and results.

Database	Molecules	Conformers	Cutoffs	Results
FDA	1856	21,850	-	1884
MNP	14,064	164,952	−6.00	19
MolPort	14,064	164,952	−6.00	145
ZINC	13,190,317	123,399,574	−6.00	149
ChemSpace2	50,181,678	250,205,463	-	50
PubChem	93,067,404	450,708,705	−7.00	98
CHEMBL25	1,752,844	23,136,925	-	128
SNP	274,363	2,928,422	−6.00	30

**Table 2:** Top 50 HDOCK server results for analyzed peptides (the gold color indicates HR1- or HR2-derived peptides, the green color is for chosen peptides and the light green and grey are for possible valuable peptides).

N.	Database	Name/Class	Id	HDOCK Score	Source
		HR1–HR2 SARS-CoV-2		−245.71	—
		HR1 SARS seq. EK1 seq.		−197.92	—
		HR1 SARS2 seq. EK1 seq.		−203.98	—
1	AVPdb	P3	AVP1841	−229.37	SARS-CoV-1 spike protein
2	AVPdb	I10L/V13L	AVP1510	−225.29	HCV non-structural protein 5A
3	AVPdb	FP4	AVP1754	−225.03	Mimetic for the SOCS protein
4	AVPdb	gH625	AVP1250	−224.33	HSV-1 H glycoprotein (gH)
5	AVPdb	BLfcin 17–31	AVP1853	−223.25	Bovine lactoferrin
6	AVPdb	—	AVP1039	−222.93	HCV envelope glycoprotein (E1, E2)
7	APD3	Cecropin A	AP00139	−219.63	Giant silk moth
8	AVPdb	—	AVP1033	−213.17	HCV envelope glycoprotein (E1, E2)
9	AVPdb	—	AVP1034	−212.76	HCV envelope glycoprotein (E1, E2)
10	AVPdb	Gly137–Arg151	AVP0430	−211.43	HSV glycoprotein (gC)
11	AVPdb	C5A	AVP1504	−210.86	HCV non-structural protein 5A
12	AVPdb	SEQ ID NO:52	AVP1463	−210.81	HCV envelope glycoprotein (E1, E2)
13	AVPdb	CL58.1	AVP1174	−208.69	Human claudin-1 (CLDN1)
14	AVPdb	GBVA4	AVP1226	−208.62	GBVA non-structural protein 5A
15	AVPdb	FP3	AVP1753	−207.82	Mimetic for the SOCS protein
16	AVPdb	SEQ ID NO:54	AVP1465	−206.96	HCV envelope glycoprotein (E1, E2)
17	AVPdb	I6L/I10L	AVP1509	−206.74	HCV non-structural protein 5A
18	AVPdb	c01	AVP0968	−206.67	Phage display
19	AVPdb	—	AVP0778	−204.47	HSV-1 B glycoprotein (gB)
20	AVPdb	—	AVP0708	−203.73	HSV-1 B glycoprotein (gB)
21	APD3	Human neutrophil peptide-3	AP00178	−203.42	Monocytes; saliva; Homo sapiens
22	AVPdb	—	AVP0793	−202.75	HSV-1 B glycoprotein (gB)
23	APD3	human neutrophil peptide-1	AP00176	−202.20	Monocytes; saliva; Homo sapiens
24	AVPdb	—	AVP0709	−201.84	HSV-1 B glycoprotein (gB)
25	AVPdb	SEQ ID NO:53	AVP1464	−199.84	HCV envelope glycoprotein (E1, E2)
26	AVPdb	CL58+2	AVP1184	−199.27	Human claudin-1 (CLDN1)
27	AVPdb	EPK209	AVP1129	−198.29	FeLV transmembrane protein (TM)
28	AVPdb	RTD3	AVP1910	−197.60	Rhesus theta-defensin
29	AVPdb	I10V	AVP1513	−197.00	HCV non-structural protein 5A
30	AVPdb	—	AVP1042	−196.98	HCV envelope glycoprotein (E1, E2)
31	APD3	Lactoferricin B	AP00026	−196.90	Bos taurus
32	APD3	Temporin A	AP00094	−196.37	European common frog
33	AVPdb	EPK210	AVP1130	−196.26	FeLV transmembrane protein (TM)
34	AVPdb	—	AVP0740	−195.56	HSV-1 B glycoprotein (gB)
35	AVPdb	SARSWW-IV	AVP0549	−193.86	SARS-CoV-1 spike protein
36	AVPdb	SEQ ID NO:62	AVP1473	−193.55	HCV envelope glycoprotein (E1, E2)
37	AVPdb	C18-p1b	AVP0966	−193.09	Phage display
38	AVPdb	CL-9	AVP1191	−193.03	Human claudin-9 (CLDN9)
39	AVPdb	E1	AVP0869	−192.85	FGF-4 signal sequence
40	AVPdb	c03	AVP0969	−192.15	Phage display
41	AVPdb	1OAN1	AVP1058	−191.92	Synthetic
42	AVPdb	GBAV5	AVP1227	−191.20	GBVA non-structural protein 5A
43	AVPdb	—	AVP0710	−190.85	HSV-1 B glycoprotein (gB)
44	AVPdb	I10A	AVP1514	−190.75	HCV non-structural protein 5A
45	AVPdb	GBVA8	AVP1230	−190.62	GBVA non-structural protein 5A
46	AVPdb	—	AVP0741	−190.33	HSV-1 B glycoprotein (gB)
47	APD3	Melittin	AP00146	−189.77	Honeybee venom
48	AVPdb	B6	AVP1087	−189.22	FGF-4 signal sequence
49	AVPdb	—	AVP0684	−188.46	HSV-1 B glycoprotein (gB)
50	AVPdb	CL58-2	AVP1183	−188.25	Human claudin-1 (CLDN1)

## Data Availability

Not applicable.

## Supplementary Materials

The supporting information can be downloaded at: https://www.mdpi.com/article/10.3390/ijms231710067/s1.

## References

[1] Lau S.K.P., Woo P.C.Y., Yip C.C.Y., Tse H., Tsoi H.W., Cheng V.C.C., Lee P., Tang B.S.F., Cheung C.H.Y., Lee R.A. (2006). Coronavirus HKU1 and other coronavirus infections in Hong Kong. J. Clin. Microbiol..

[2] Chan J.F.W., Kok K.H., Zhu Z., Chu H., To K.K.W., Yuan S., Yuen K.Y. (2020). Genomic characterization of the 2019 novel human-pathogenic coronavirus isolated from a patient with atypical pneumonia after visiting Wuhan. Emerg. Microbes Infect..

[3] Chan J.F.W., Lau S.K.P., To K.K.W., Cheng V.C.C., Woo P.C.Y., Yuen K.Y. (2015). Middle East Respiratory Syndrome Coronavirus: Another Zoonotic Betacoronavirus Causing SARS-Like Disease. Clin. Microbiol. Rev..

[4] Cheng V.C.C., Lau S.K.P., Woo P.C.Y., Yuen K.Y. (2007). Severe acute respiratory syndrome coronavirus as an agent of emerging and reemerging infection. Clin. Microbiol. Rev..

[5] Chan J.F.W., To K.K.W., Tse H., Jin D.Y., Yuen K.Y. (2013). Interspecies transmission and emergence of novel viruses: Lessons from bats and birds. Trends Microbiol..

[6] Zhou P., Yang X.L., Wang X.G., Hu B., Zhang L., Zhang W., Si H.R., Zhu Y., Li B., Huang C.L. (2020). A pneumonia outbreak associated with a new coronavirus of probable bat origin. Nature.

[7] Gorbalenya A.E., Baker S.C., Baric R.S., de Groot R.J., Drosten C., Gulyaeva A.A., Haagmans B.L., Lauber C., Leontovich A.M., Neuman B.W. (2020). The species Severe acute respiratory syndrome-related coronavirus: Classifying 2019-nCoV and naming it SARS-CoV-2. Nat. Microbiol..

[8] McCarty T.R., Hathorn K.E., Redd W.D., Rodriguez N.J., Zhou J.C., Bazarbashi A.N., Njie C., Wong D., Trinh Q.D., Shen L. (2021). How Do Presenting Symptoms and Outcomes Differ by Race/Ethnicity Among Hospitalized Patients With Coronavirus Disease 2019 Infection? Experience in Massachusetts. Clin. Infect. Dis..

[9] Martin W.R., Cheng F.X. (2020). Repurposing of FDA-Approved Toremifene to Treat COVID-19 by Blocking the Spike Glycoprotein and NSP14 of SARS-CoV-2. J. Proteome Res..

[10] Barnes C.O., Jette C.A., Abernathy M.E., Dam K.A., Esswein S.R., Gristick H.B., Malyutin A.G., Sharaf N.G., Huey-Tubman K.E., Lee Y.E. (2020). Structural classification of neutralizing antibodies against the SARS-CoV-2 spike receptor-binding domain suggests vaccine and therapeutic strategies. bioRxiv.

[11] Creech C.B., Walker S.C., Samuels R.J. (2021). SARS-CoV-2 vaccines. JAMA.

[12] Baranov P.V., Henderson C.M., Anderson C.B., Gesteland R.F., Atkins J.F., Howard M.T. (2005). Programmed ribosomal frameshifting in decoding the SARS-CoV genome. Virology.

[13] Wrapp D., Wang N., Corbett K.S., Goldsmith J.A., Hsieh C.-L., Abiona O., Graham B.S., McLellan J.S. (2020). Cryo-EM structure of the 2019-nCoV spike in the prefusion conformation. Science.

[14] Ling R., Dai Y., Huang B., Huang W., Yu J., Lu X., Jiang Y. (2020). In silico design of antiviral peptides targeting the spike protein of SARS-CoV-2. Peptides.

[15] Cai Y., Zhang J., Xiao T., Peng H., Sterling S.M., Walsh R.M., Rawson S., Rits-Volloch S., Chen B. (2020). Distinct conformational states of SARS-CoV-2 spike protein. Science.

[16] Xia S., Liu M., Wang C., Xu W., Lan Q., Feng S., Qi F., Bao L., Du L., Liu S. (2020). Inhibition of SARS-CoV-2 (previously 2019-nCoV) infection by a highly potent pan-coronavirus fusion inhibitor targeting its spike protein that harbors a high capacity to mediate membrane fusion. Cell Res..

[17] Liu S., Xiao G., Chen Y., He Y., Niu J., Escalante C.R., Xiong H., Farmar J., Debnath A.K., Tien P. (2004). Interaction between heptad repeat 1 and 2 regions in spike protein of SARS-associated coronavirus: Implications for virus fusogenic mechanism and identification of fusion inhibitors. Lancet.

[18] Lu L., Liu Q., Zhu Y., Chan K.-H., Qin L., Li Y., Wang Q., Chan J.F.-W., Du L., Yu F. (2014). Structure-based discovery of Middle East respiratory syndrome coronavirus fusion inhibitor. Nat. Commun..

[19] Xia S., Zhu Y., Liu M., Lan Q., Xu W., Wu Y., Ying T., Liu S., Shi Z., Jiang S. (2020). Fusion mechanism of 2019-nCoV and fusion inhibitors targeting HR1 domain in spike protein. Cell. Mol. Immunol..

[20] Zhu Y., Yu D., Yan H., Chong H., He Y. (2020). Design of potent membrane fusion inhibitors against SARS-CoV-2, an emerging coronavirus with high fusogenic activity. J. Virol..

[21] Xia S., Yan L., Xu W., Agrawal A.S., Algaissi A., Tseng C.-T.K., Wang Q., Du L., Tan W., Wilson I.A. (2019). A pan-coronavirus fusion inhibitor targeting the HR1 domain of human coronavirus spike. Sci. Adv..

[22] Lei S., Zheng R., Zhang S., Wang S., Chen R., Sun K., Zeng H., Zhou J., Wei W. (2021). Global patterns of breast cancer incidence and mortality: A population-based cancer registry data analysis from 2000 to 2020. Cancer Commun..

[23] LaBonte J., Lebbos J., Kirkpatrick P. (2003). Enfuvirtide. Nat. Rev. Drug Discov..

[24] Chu L.H.M., Chan S.H., Tsai S.N., Wang Y., Cheng C.H.K., Wong K.B., Waye M.M.Y., Ngai S.M. (2008). Fusion core structure of the severe acute respiratory syndrome coronavirus (SARS-CoV): In search of potent SARS-CoV entry inhibitors. J. Cell. Biochem..

[25] Singh R., Bhardwaj V.K., Sharma J., Purohit R., Kumar S. (2022). In-silico evaluation of bioactive compounds from tea as potential SARS-CoV-2 nonstructural protein 16 inhibitors. J. Tradit. Complement. Med..

[26] Singh R., Bhardwaj V.K., Sharma J., Kumar D., Purohit R. (2021). Identification of potential plant bioactive as SARS-CoV-2 Spike protein and human ACE2 fusion inhibitors. Comput. Biol. Med..

[27] Chauhan M., Bhardwaj V.K., Kumar A., Kumar V., Kumar P., Enayathullah M.G., Thomas J., George J., Kumar B.K., Purohit R. (2022). Theaflavin 3-gallate inhibits the main protease (M(pro)) of SARS-CoV-2 and reduces its count in vitro. Sci. Rep..

[28] Kashyap P., Bhardwaj V.K., Chauhan M., Chauhan V., Kumar A., Purohit R., Kumar A., Kumar S. (2022). A ricin-based peptide BRIP from Hordeum vulgare inhibits M(pro) of SARS-CoV-2. Sci. Rep..

[29] Bhardwaj V.K., Singh R., Sharma J., Rajendran V., Purohit R., Kumar S. (2021). Identification of bioactive molecules from tea plant as SARS-CoV-2 main protease inhibitors. J. Biomol. Struct. Dyn..

[30] Singh R., Bhardwaj V.K., Purohit R. (2021). Potential of turmeric-derived compounds against RNA-dependent RNA polymerase of SARS-CoV-2: An in-silico approach. Comput. Biol. Med..

[31] Bhardwaj V.K., Singh R., Sharma J., Rajendran V., Purohit R., Kumar S. (2021). Bioactive Molecules of Tea as Potential Inhibitors for RNA-Dependent RNA Polymerase of SARS-CoV-2. Front. Med..

[32] Singh R., Bhardwaj V.K., Das P., Purohit R. (2021). A computational approach for rational discovery of inhibitors for non-structural protein 1 of SARS-CoV-2. Comput. Biol. Med..

[33] Bhardwaj V.K., Singh R., Das P., Purohit R. (2021). Evaluation of acridinedione analogs as potential SARS-CoV-2 main protease inhibitors and their comparison with repurposed anti-viral drugs. Comput. Biol. Med..

[34] Sharma J., Kumar Bhardwaj V., Singh R., Rajendran V., Purohit R., Kumar S. (2021). An in-silico evaluation of different bioactive molecules of tea for their inhibition potency against non structural protein-15 of SARS-CoV-2. Food Chem..

[35] Singh R., Bhardwaj V.K., Das P., Bhattacherjee D., Zyryanov G.V., Purohit R. (2022). Benchmarking the ability of novel compounds to inhibit SARS-CoV-2 main protease using steered molecular dynamics simulations. Comput. Biol. Med..

[36] Floresta G., Zagni C., Gentile D., Patamia V., Rescifina A. (2022). Artificial Intelligence Technologies for COVID-19 De Novo Drug Design. Int. J. Mol. Sci..

[37] Cardullo N., Catinella G., Floresta G., Muccilli V., Rosselli S., Rescifina A., Bruno M., Tringali C. (2019). Synthesis of Rosmarinic Acid Amides as Antioxidative and Hypoglycemic Agents. J. Nat. Prod..

[38] Floresta G., Amata E., Gentile D., Romeo G., Marrazzo A., Pittalà V., Salerno L., Rescifina A. (2019). Fourfold Filtered Statistical/Computational Approach for the Identification of Imidazole Compounds as HO-1 Inhibitors from Natural Products. Mar. Drugs.

[39] Floresta G., Pistarà V., Amata E., Dichiara M., Damigella A., Marrazzo A., Prezzavento O., Punzo F., Rescifina A. (2018). Molecular modeling studies of pseudouridine isoxazolidinyl nucleoside analogues as potential inhibitors of the pseudouridine 5ʹ-monophosphate glycosidase. Chem. Biol. Drug Des..

[40] Floresta G., Patamia V., Gentile D., Molteni F., Santamato A., Rescifina A., Vecchio M. (2020). Repurposing of FDA-Approved Drugs for Treating Iatrogenic Botulism: A Paired 3D-QSAR/Docking Approach. ChemMedChem.

[41] Floresta G., Gentile D., Perrini G., Patamia V., Rescifina A. (2019). Computational Tools in the Discovery of FABP4 Ligands: A Statistical and Molecular Modeling Approach. Mar. Drugs.

[42] Floresta G., Rescifina A., Abbate V. (2019). Structure-Based Approach for the Prediction of Mu-opioid Binding Affinity of Unclassified Designer Fentanyl-Like Molecules. Int. J. Mol. Sci..

[43] Gentile D., Floresta G., Patamia V., Chiaramonte R., Mauro G.L., Rescifina A., Vecchio M. (2020). An Integrated Pharmacophore/Docking/3D-QSAR Approach to Screening a Large Library of Products in Search of Future Botulinum Neurotoxin A Inhibitors. Int. J. Mol. Sci..

[44] Xia S., Lan Q., Zhu Y., Wang C., Xu W., Li Y., Wang L., Jiao F., Zhou J., Hua C. (2021). Structural and functional basis for pan-CoV fusion inhibitors against SARS-CoV-2 and its variants with preclinical evaluation. Signal Transduct. Target. Ther..

[45] Simmaco M., Mignogna G., Canofeni S., Miele R., Mangoni M.L., Barra D. (1996). Temporins, antimicrobial peptides from the European red frog Rana temporaria. Eur. J. Biochem..

[46] Mangoni M. (2006). Temporins, anti-infective peptides with expanding properties. Cell. Mol. Life Sci. CMLS.

[47] Swithenbank L., Cox P., Harris L.G., Dudley E., Sinclair K., Lewis P., Cappiello F., Morgan C. (2020). Temporin A and Bombinin H2 Antimicrobial Peptides Exhibit Selective Cytotoxicity to Lung Cancer Cells. Scientifica.

[48] Liu X., Huang Y., Cheng M., Pan L., Si Y., Li G., Niu Y., Zhao L., Zhao J., Li X. (2013). Screening and rational design of hepatitis C virus entry inhibitory peptides derived from GB virus A NS5A. J. Virol..

[49] Yang C., Pan X., Xu X., Cheng C., Huang Y., Li L., Jiang S., Xu W., Xiao G., Liu S. (2020). Salvianolic acid C potently inhibits SARS-CoV-2 infection by blocking the formation of six-helix bundle core of spike protein. Signal Transduct. Target. Ther..

[50] Rowley D.C., Hansen M.S., Rhodes D., Sotriffer C.A., Ni H., McCammon J.A., Bushman F.D., Fenical W. (2002). Thalassiolins A–C: New marine-derived inhibitors of HIV cDNA integrase. Bioorg. Med. Chem..

[51] Regalado E.L., Rodríguez M., Menéndez R., Concepción Á.A., Nogueiras C., Laguna A., Rodríguez A.A., Williams D.E., Lorenzo-Luaces P., Valdés O. (2009). Repair of UVB-damaged skin by the antioxidant sulphated flavone glycoside thalassiolin B isolated from the marine plant Thalassia testudinum Banks ex König. Mar. Biotechnol..

[52] Stewart J.J. (2004). Optimization of parameters for semiempirical methods IV: Extension of MNDO, AM1, and PM3 to more main group elements. J. Mol. Modeling.

[53] Yan Y., Tao H., He J., Huang S.-Y. (2020). The HDOCK server for integrated protein–protein docking. Nat. Protoc..

[54] Krieger E., Vriend G. (2014). YASARA View—molecular graphics for all devices—from smartphones to workstations. Bioinformatics.

[55] Krieger E., Koraimann G., Vriend G. (2002). Increasing the precision of comparative models with YASARA NOVA—A self-parameterizing force field. Proteins Struct. Funct. Bioinform..

[56] Xu Y., Lou Z., Liu Y., Pang H., Tien P., Gao G.F., Rao Z. (2004). Crystal structure of severe acute respiratory syndrome coronavirus spike protein fusion core. J. Biol. Chem..

[57] Duan Y., Wu C., Chowdhury S., Lee M.C., Xiong G., Zhang W., Yang R., Cieplak P., Luo R., Lee T. (2003). A point-charge force field for molecular mechanics simulations of proteins based on condensed-phase quantum mechanical calculations. J. Comput. Chem..

[58] Krieger E., Dunbrack R.L., Hooft R.W., Krieger B. (2012). Assignment of protonation states in proteins and ligands: Combining pK a prediction with hydrogen bonding network optimization. Computational Drug Discovery and Design.

[59] Krieger E., Nielsen J.E., Spronk C.A., Vriend G. (2006). Fast empirical pKa prediction by Ewald summation. J. Mol. Graph. Model..

[60] Maier J., Martinez C., Kasavajhala K., Wickstrom L., Hauser K., Simmerling C. (2015). ff14SB: Improving the Accuracy of Protein Side Chain and Backbone Parameters from ff99SB. J. Chem. Theory Comput..

[61] Wang J., Wolf R.M., Caldwell J.W., Kollman P.A., Case D.A. (2004). Development and testing of a general amber force field. J. Comput. Chem..

[62] Jakalian A., Jack D.B., Bayly C.I. (2002). Fast, efficient generation of high-quality atomic charges. AM1-BCC model: II. Parameterization and validation. J. Comput. Chem..

[63] Hornak V., Abel R., Okur A., Strockbine B., Roitberg A., Simmerling C. (2006). Comparison of multiple Amber force fields and development of improved protein backbone parameters. Proteins Struct. Funct. Bioinform..

[64] Essmann U., Perera L., Berkowitz M.L., Darden T., Lee H., Pedersen L.G. (1995). A smooth particle mesh Ewald method. J. Chem. Phys..

[65] Galimberti M., Barbera V., Guerra S., Bernardi A. (2017). Facile functionalization of sp2 carbon allotropes with a biobased Janus molecule. Rubber Chem. Technol..

[66] Krieger E., Vriend G. (2015). New ways to boost molecular dynamics simulations. J. Comput. Chem..

