# Supplementary material for: Targeting the SARS-CoV-2 HR1 with Small Molecules as Inhibitors of the Fusion Process
Source: Int J Mol Sci. 2022 Sep 3;23(17):10067. doi: 10.3390/ijms231710067 (PMC9456533; doi:10.3390/ijms231710067)
Supplement: Supplementary file 1 [file ijms-23-10067-s001.zip › ijms-1864044-supplementary.pdf]

International Journal of
Molecular Sciences

MDPI

Supplementary material

Targeting the SARS-CoV-2 HR1 with Small Molecules as Inhibitors of the Fusion Process

Davide Gentile, Alessandro Coco, Vincenzo Patamia, Chiara Zagni, Giuseppe Floresta and Antonio Rescifina *

Dipartimento di Scienze del Farmaco e della Salute, Università di Catania, Viale A. Doria 6, 95125 Catania, Italy
* Correspondence: arescifina@unict.it

Table of content

Scheme S1	2
Figure S1	3
Figure S2	3
Figure S3	4
Figure S4	4
Figure S5	5
Figure S6	5
Figure S7	6
Figure S8	6
Figure S9	7
Figure S10	7
Table S1	8

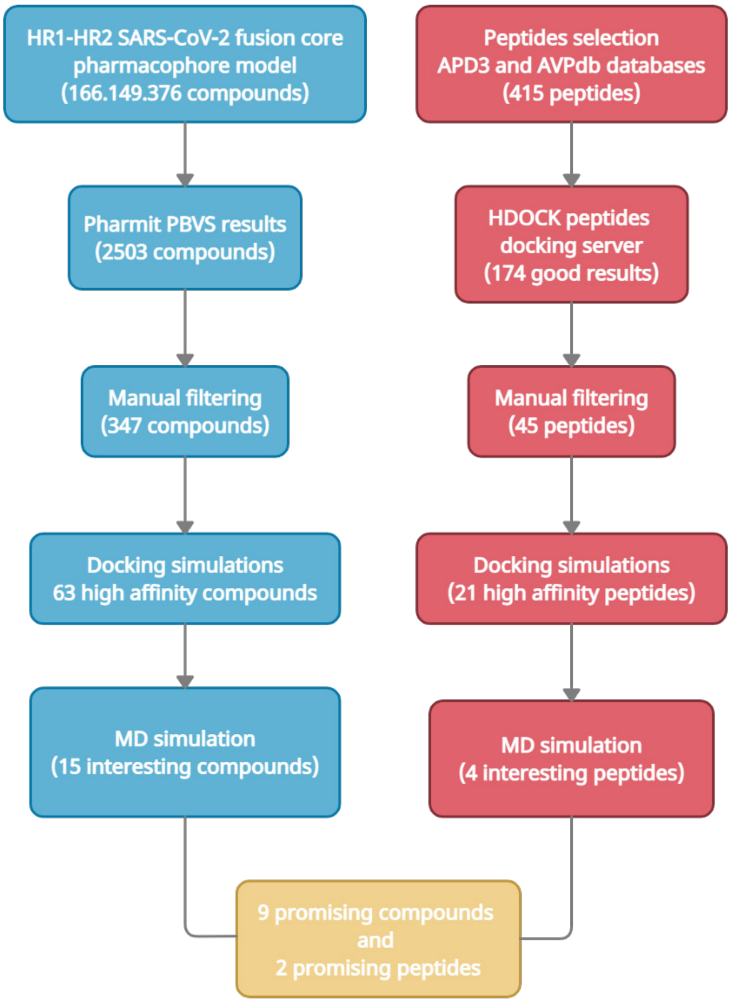

Scheme S1. Used workflow for the *in-silico* screening.

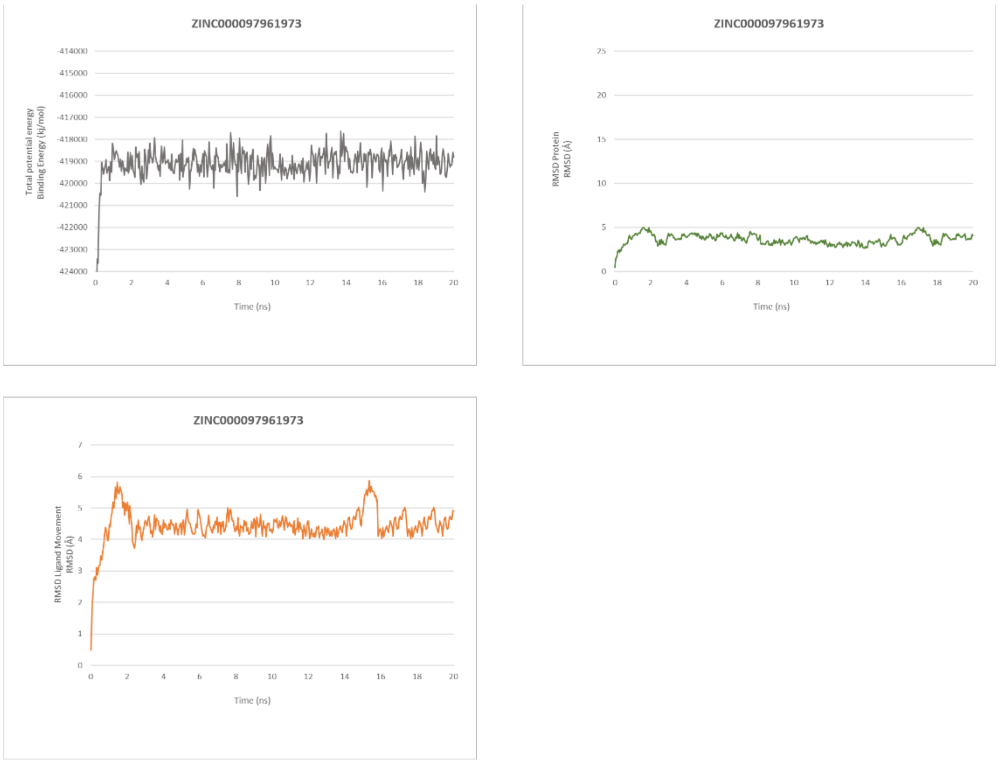

Figure S1. ZINC000097961973@HR1 domain. Total energy (up-left), RMSDs of protein (up-right) and RMSDs of ligand (down-left).

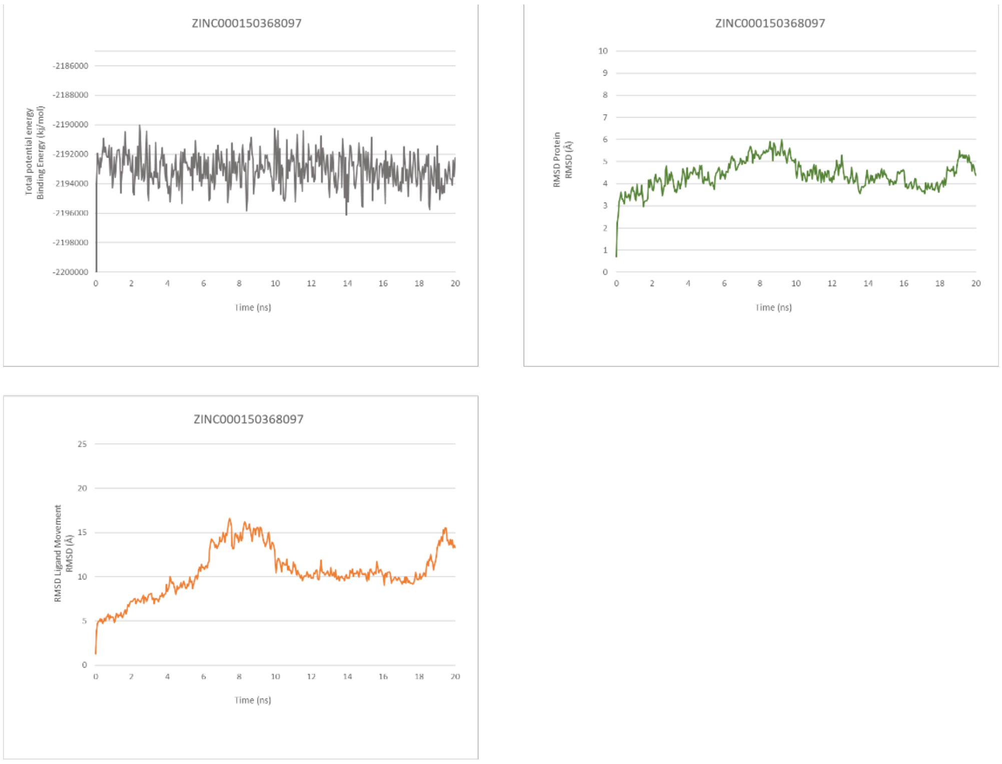

Figure S2. ZINC000150368097@HR1 domain. Total energy (up-left), RMSDs of protein (up-right) and RMSDs of ligand (down-left).

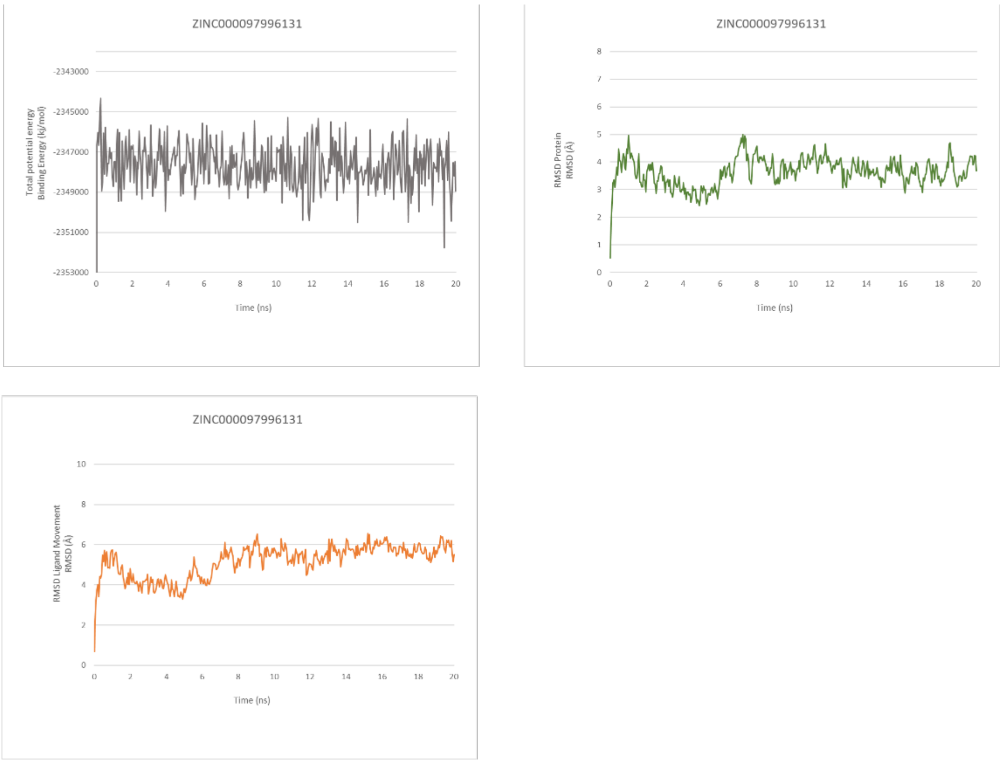

Figure S3. ZINC000097996131@HR1 domain. Total energy (up-left), RMSDs of protein (up-right) and RMSDs of ligand (down-left).

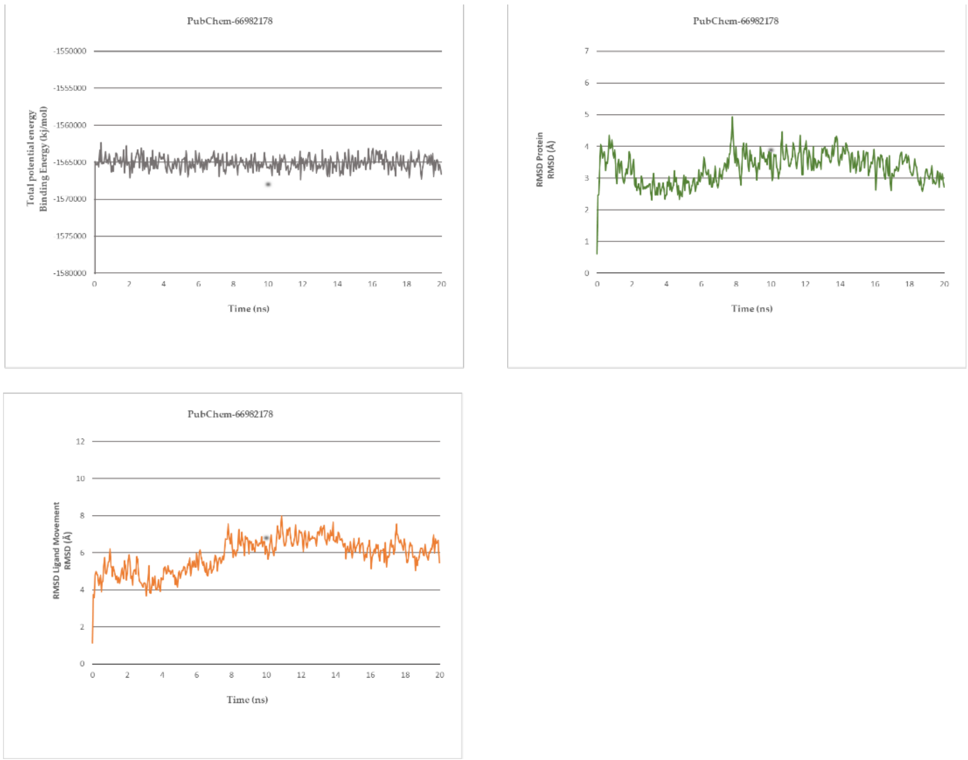

Figure S4. PubChem-66982178@HR1 domain. Total energy (up-left), RMSDs of protein (up-right) and RMSDs of ligand (down-left).

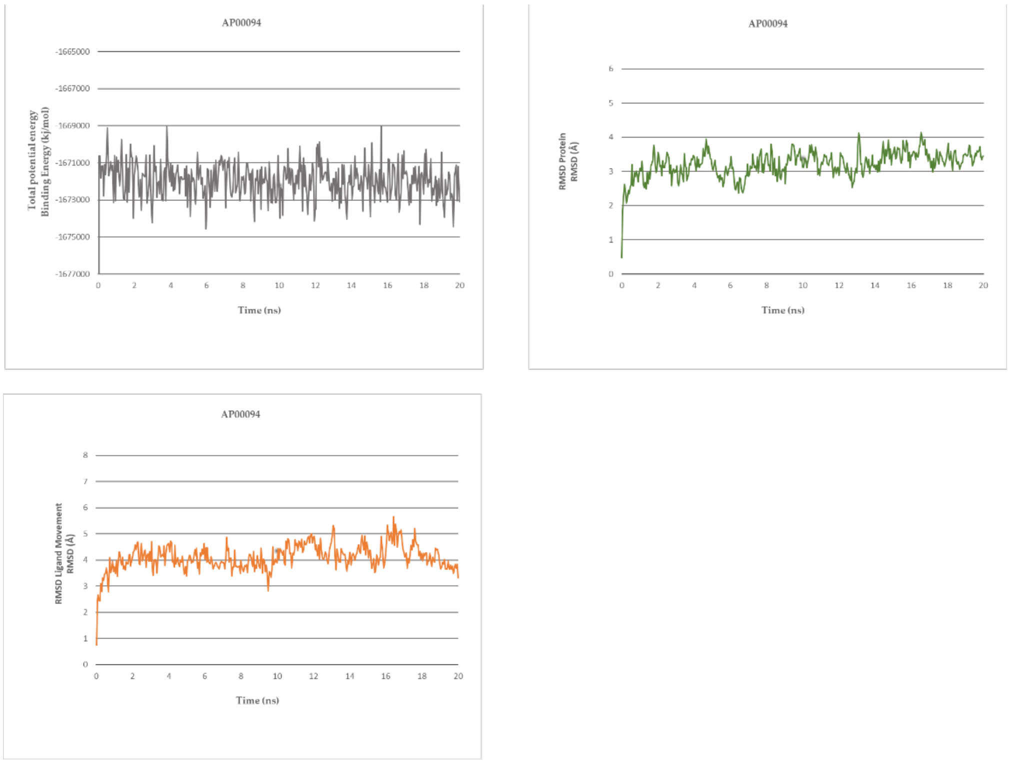

Figure S5. AP00094@HR1 domain. Total energy (up-left), RMSDs of protein (up-right) and RMSDs of ligand (down-left).

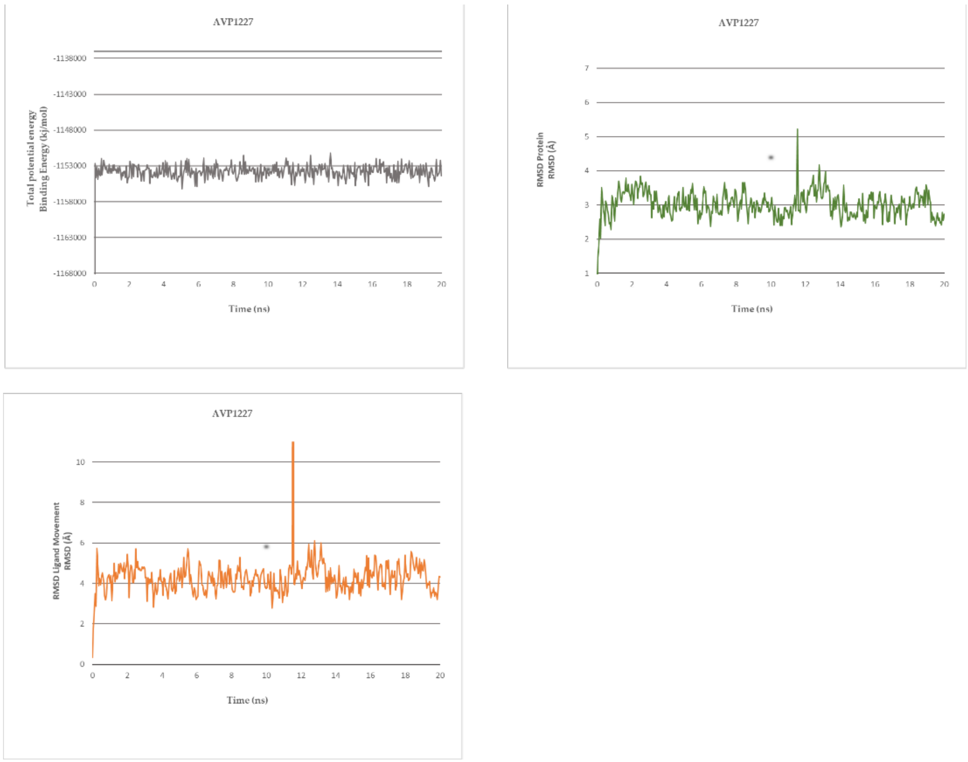

Figure S6. AVP1227@HR1 domain. Total energy (up-left), RMSDs of protein (up-right) and RMSDs of ligand (down-left).

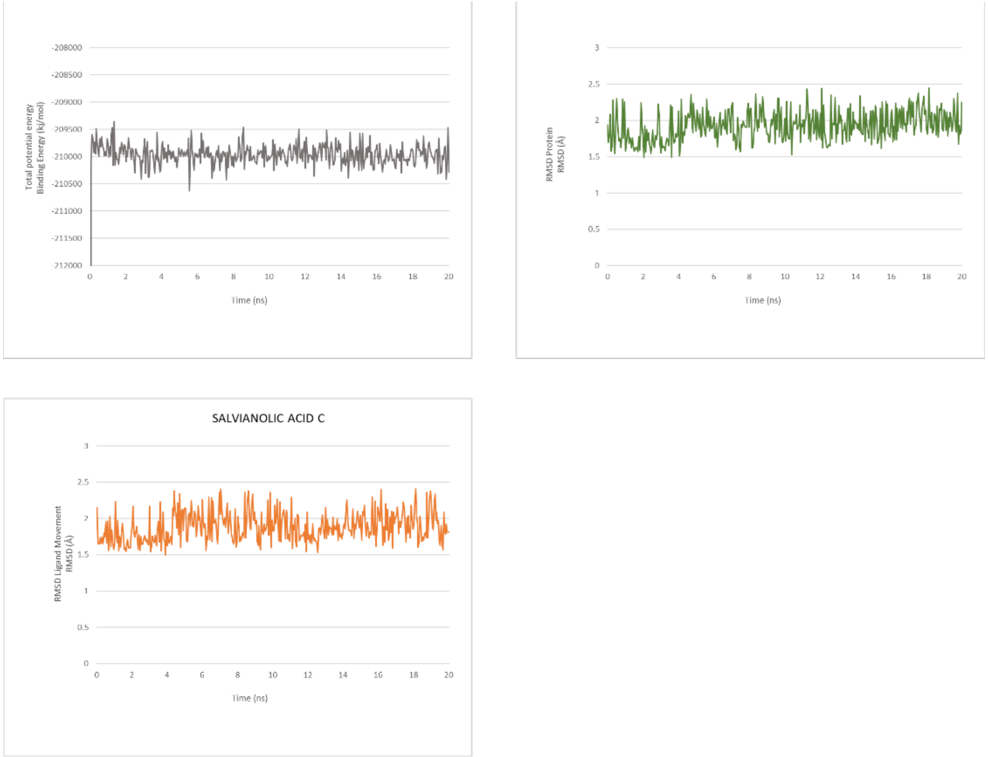

Figure S7. Sal C@HR1 domain. Total energy (up-left), RMSDs of protein (up-right) and RMSDs of ligand (down-left).

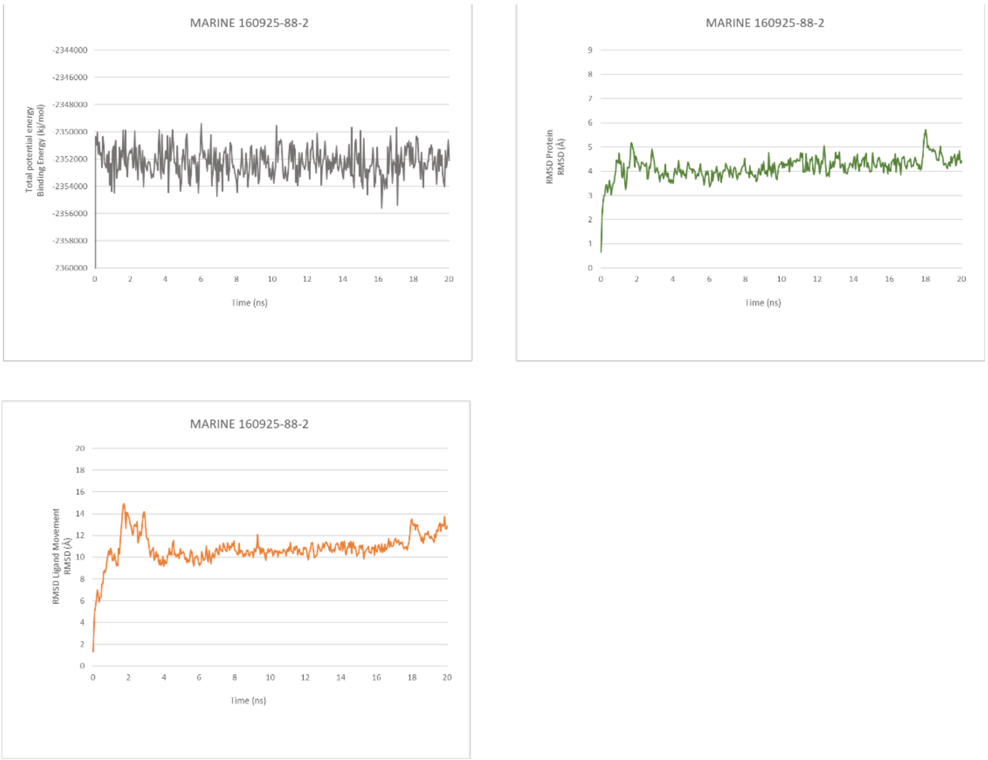

Figure S8. Marine-160925-88-2@HR1 domain. Total energy (up-left), RMSDs of protein (up-right) and RMSDs of ligand (down-left).

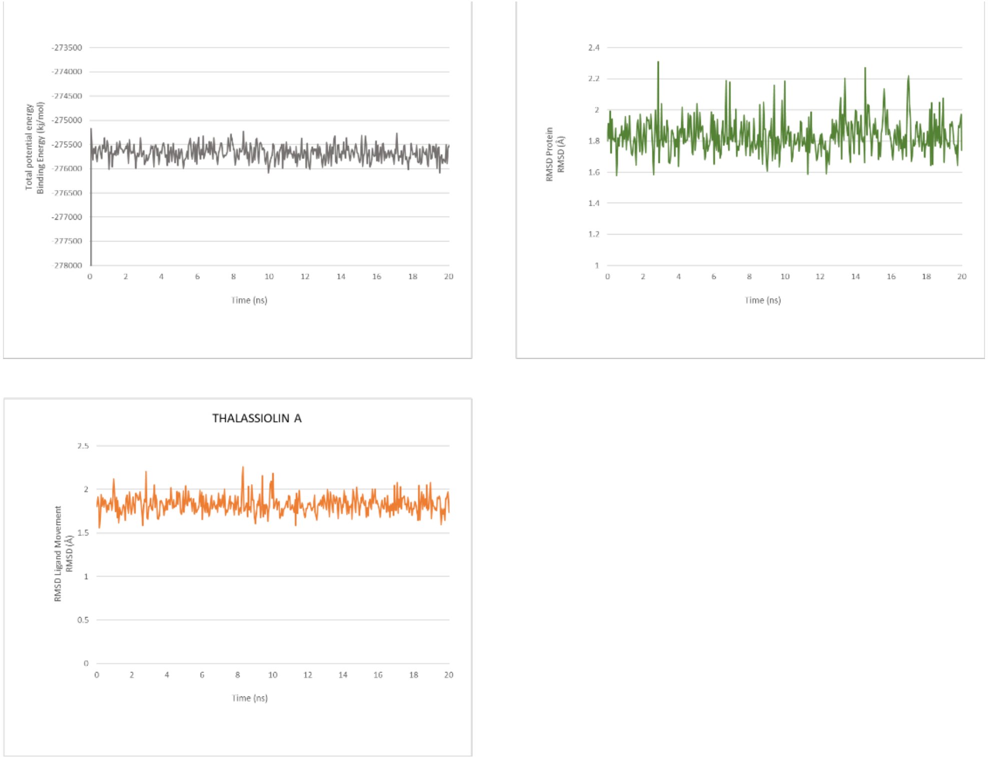

Figure S9. Thalassiolin A@HR1 domain. Total energy (up-left), RMSDs of protein (up-right) and RMSDs of ligand (down-left).

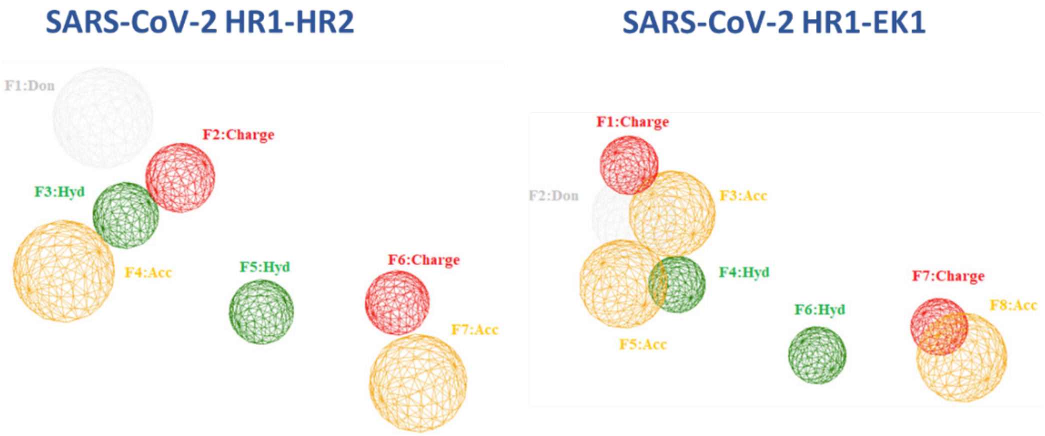

Figure S10. Main interactions described in the pharmacophore model of HR1-HR2 and HR1-EK1.

Table S1. AutodockLGA simulation results for best-scoring compounds.

N.	Name	Structure	ΔG Auto-dock4 kcal/mol
1	NF 023 hydrate PubChem-24278597	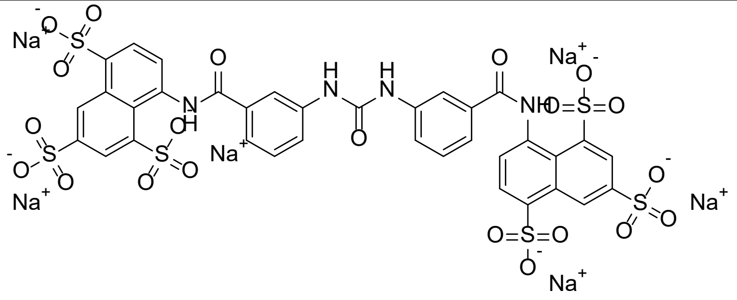	−12.03
2	ZINC000097961973	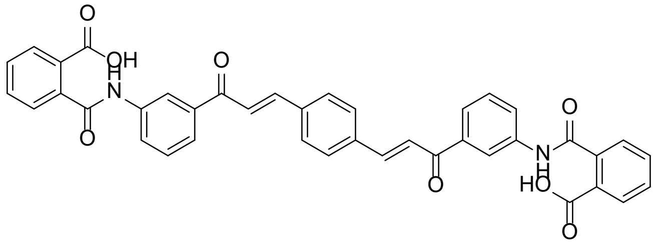	−10.73
3	ZINC000150368097	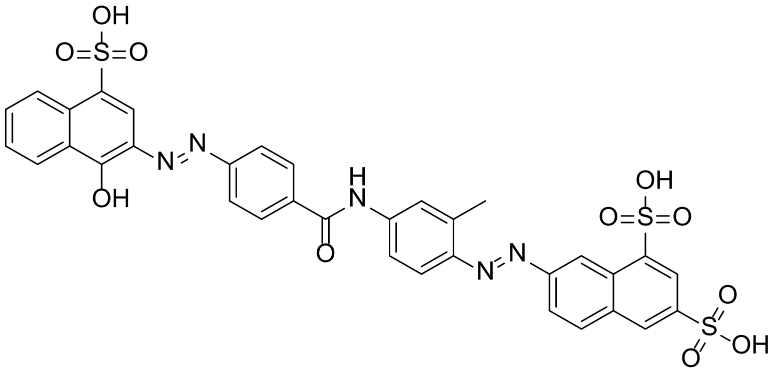	−10.68
4	ZINC000169332064	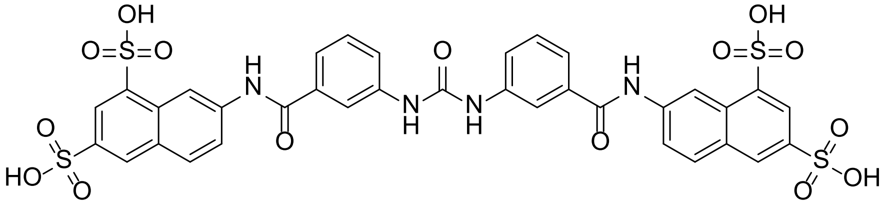	−10.59
5	ZINC000097996131	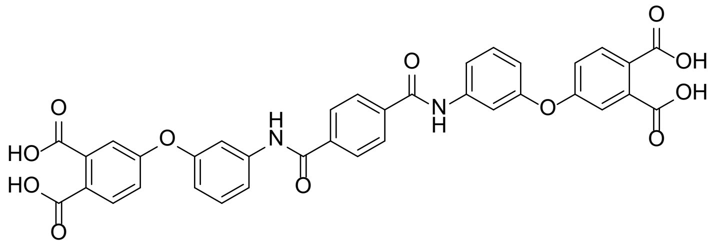	−10.38

6	ZINC000150346512	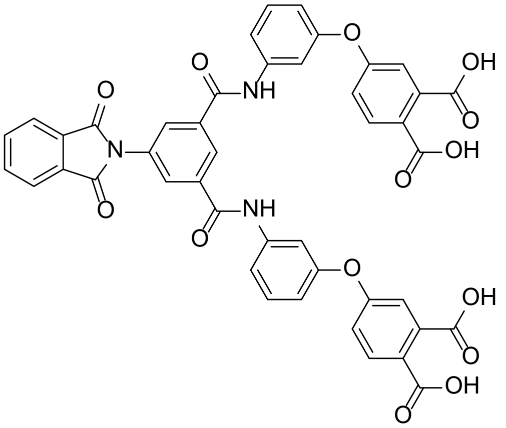	–10.34
7	ZINC000150631141	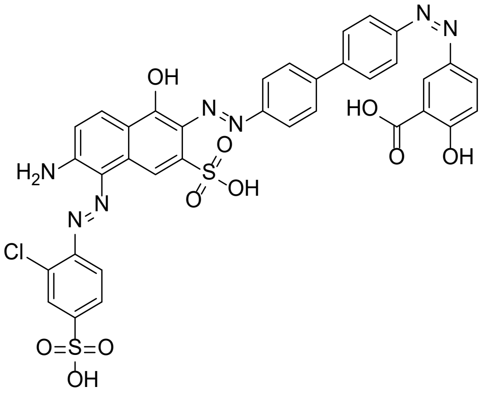	–10.31
8	Marine-160925-88-2	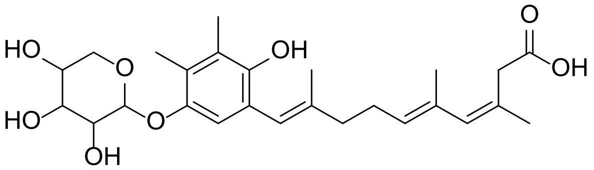	–10.21
9	PubChem-66982178	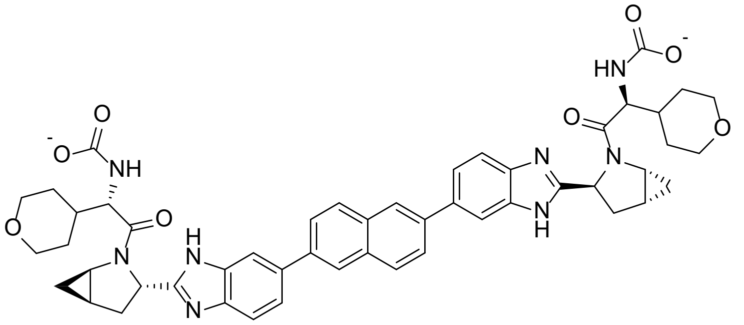	–10.12
10	ZINC000150485530	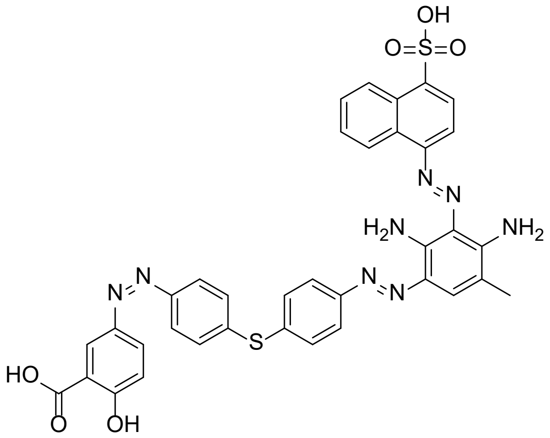	–10.04

11	PubChem-24742078	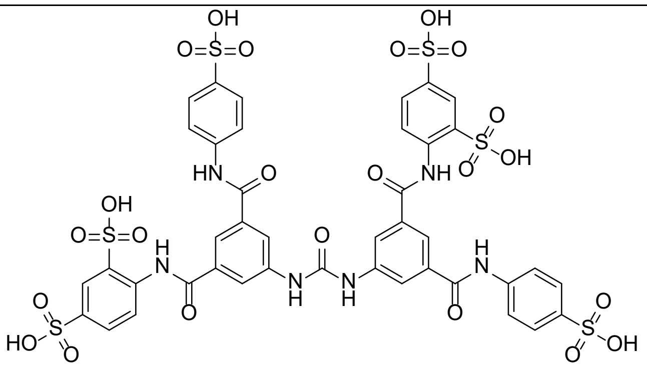	−9.94
12	Thalassiolin A	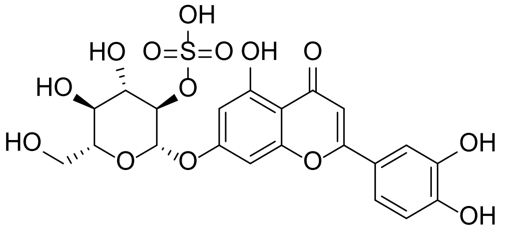	−9.94
13	ADS-J1@SARS-CoV-1 fusion core	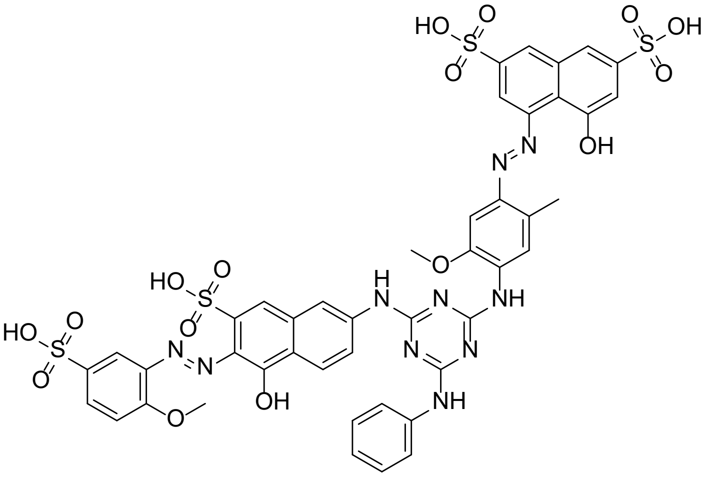	−9.76
14	ZINC263603439	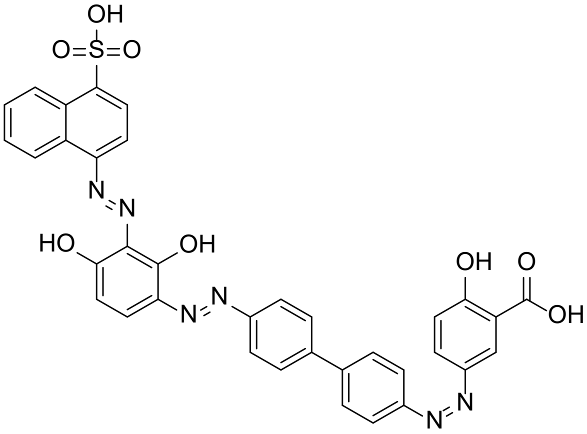	−9.73

15	ADS-J1@SARS-CoV-2 fusion core	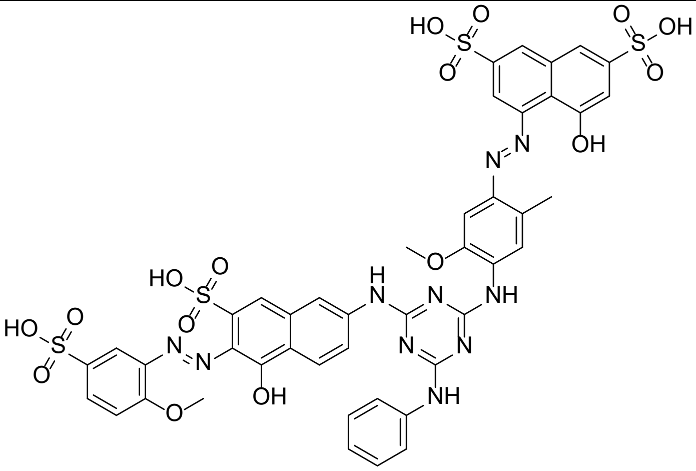	–8.80
16	Salvianolic acid C	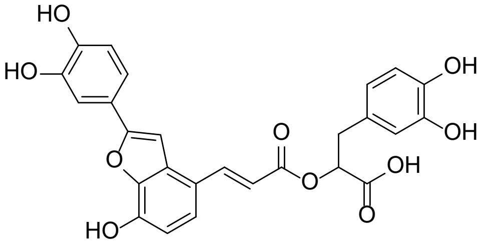	–8.75
17	PubChem-10169258	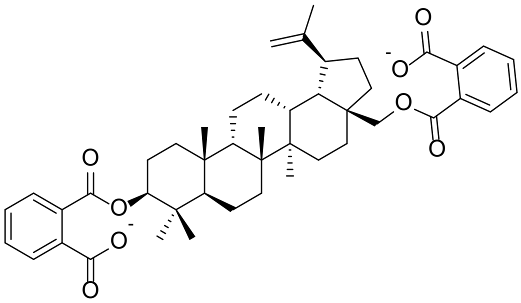	–8.26
